# Supplementary material for: Placebos in primary care? a nominal group study explicating UK GP and patient views of six theoretically plausible models of placebo practice
Source: BMJ Open. 2020 Feb 18;10(2):e032524. doi: 10.1136/bmjopen-2019-032524 (PMC7044897; doi:10.1136/bmjopen-2019-032524)
Supplement: Supplementary data [file bmjopen-2019-032524supp001.pdf]

Appendix 1: Summary of Themes

Theme	Definition	Example quote
Consultation and clinical practice behaviours	General talk which is not necessarily specific to placebogenic practice but which dictates the models of consultation and also broader consideration about working life of a GP.	*I think very few medications I prescribe actually have the best chance of reducing anyone's symptoms in a short time. The majority of medications I'm prescribing are preventative, and that's when you often have to do the sales job, because they're not going to have any change in their symptoms but you just try and give them what's needed to treat them, or I have done. So I think the preventative thing is quite interesting. But I would use the technique at times.*<br><br>(GP Group 3)
Patient perspectives on GP behaviour	General talk about the need for GPs to treat patients as individuals and how GPs should respect patients, e.g. by how they listen and talk to them. Not linked to placebogenic practice. Mainly derived from general talk in the patient groups about past experiences with GPs.	*Yes, because I think that all treatment should be a holistic approach, in that you have got to treat the whole person. It is all very well treating one condition, but sometimes when there are multiple conditions, or it is one condition that has multiple effects, you need to look at the actual person, and to me, very much, this was the approach of looking at one person, and making them feel … and they are taking part in it. And I think that is important.*<br><br>(Patient Group 1)
Placebo models	Talk that alludes to how participants think placebos might operate to generate effects on patients. How do they work, if indeed they do work? Not specifically linked to a particular placebogenic practice under discussion, rather this is more general talk	*Well this is where the placebo effect comes in – the expectation*<br><br>(Patient Group 4)

	about the mechanisms thought to possibly underpin placebo effects.	
Placebogenic practice – health system and medico-legal framework consideration	Aspects of the wider health system that are relevant to the acceptability of placebogenic practice e.g. GMC rules or health targets or legal framework of clinical practice	*And again, there's the risks, especially if there's, you know, it impaired their function and they had an accident or, you know, there's risks to not telling people about potential side effects.*<br><br>(GP Group 1)
Placebogenic practice – honesty, ethical practice and disclosure	Talk about the acceptability and ethical issues around honesty, deception, and complete/incomplete disclosure. About the information that "should" be provided to patients, the information that patients want, and why this is seen as important. Talk about the moral imperative for full information and informed consent, as well as occasional situations where these things are deemed non-essential. Includes talk about the circumstances in which some dishonesty or incomplete disclosure might be tolerated.	*That's unethical. So, if you've got a 1 in 10 chance that actually taking this medication is going to cause you some significant harm, we've got to be completely open about this*.<br><br>(GP Group 2)
Placebogenic practice – patient considerations	Aspects of the patient (e.g. characteristics, beliefs, medical condition, clinical history) that are relevant to considering how acceptable a placebogenic practice is. Does NOT include talk about how it is important to treat patients as individuals or to know one's patient very well (this is coded within placebogenic practice - therapeutic encounter considerations).	*The only observation I made just really when we started talking is that patients who may have that suspicious mind-set about drugs anyway tend to research side effect pretty effectively anyway. Again, if you know your patient you kind of reinforce the patient information leaflet about all the bad things that might happen.*<br><br>(GP Group 4)
Placebogenic practice – patient outcomes	The effects that participants think might flow from a particular placebogenic practice. These are effects on the patient as an individual (as opposed to their behaviour in	*F1: It's interesting because it could be that some people might think, oh you know, she cares about me and she wants to see me more, that's a positive thing,*

	relation to consulting and the doctor-patient encounter/relationship). Includes potential benefits and harms, of all types (e.g. includes psychological, physical, financial considerations). Also includes talk about the possible lack of effects of a placebogenic practice - i.e. when participants think that it would not work.	*F2: But it might also be a sign of added interest from –*<br><br>*F1: Yes, staying on top of it, you know, wanting to be on your shoulders, not fobbed off with a diary.*<br><br>(Patient Group 2)
Placebogenic practice – practitioner consequences	The effects that participants this might stem from a particular placebogenic practice. These are effects on the doctor as an individual and could include potential benefits and harms of all types.	[Quote from 'Monitoring' Scenario]<br>*I suppose I would use it for a few reasons as well. One would be both to better inform myself, because often patients come in with these vague symptoms and you're never quite sure what's actually happening, because they don't almost really know themselves. So actually getting them to sit down and actually write it out sometimes is very helpful, again, for them to establish, actually, this is the pattern of work. Maybe it isn't as bad as I thought it was because, of course, patients will often think the worst. So that can be really helpful. I think I probably also have used it as a procrastination technique every now and again, because I do think a medication will work but it just needs a bit more time.*<br><br>(GP Group 3)
Placebogenic practice – the practitioner considerations	Properties, characteristics, etc. of the practitioner that are relevant to considering the acceptability of a placebogenic practice. Includes discussion related to the practitioner's status, their qualifications and expertise, and their intentions	*Male 3: I'm not certain that last statement or sentence, 'if you tell him about it he'll be more likely to suffer from them' is true. I think there's a small cohort of patients where you tell them the side effects they will get it, and I could name a few patients where if I*

	guiding the placebogenic practice.	*say you're going to get cough with giving an ace inhibitor, they will cough. So those are the ones where arguably actually I would not tell them that you might get a cough about it. But I think on the whole patients –*<br><br>*Male 1: Would you be happy for one of your patients to come back to you nine months later and say, "I've had this cough for nine months," but they weren't aware it was side effect and they've had to live with that for nine months because you hadn't told them about it?*<br><br>*Male 3: I'd happily live with that, it's only a bit of a dry cough, it's not the end of the world, it's not going to kill –*<br><br>(GP Group 1)
Placebogenic practice – therapeutic encounter consequences	Talk about consequences of the placebogenic practice for the therapeutic encounter, the doctor-patient relationship, and future consultations. Includes talk about both positive and negative consequences.	*To me, it depends upon the frequency and the severity of the side effects. Because if they're rare and minor I would be completely comfortable with it, if they're serious or very frequent I'd be uncomfortable with it because you risk loss of trust, I think, from your patient if you don't tell them.*<br><br>(GP Group 1)
Placebogenic practice – therapeutic encounter considerations	Issues about the doctor-patient relationship in general and the consultation in particular that are deemed relevant to considering the acceptability of a placebogenic practice. Incudes discussion of the therapeutic encounter and its characteristics and how these might influence whether a particular practice is acceptable. Also includes	*I was going to say the same thing and it's the thing that is the doctor is the drug relationship, where you are using your ongoing built-up trust with the patient to have this effect, but if the effect doesn't actually happen or if the patient doesn't derive the benefit what then happens is you've lost some of that capital of the relationship. So*

	discussion of how a placebogenic practice itself represents a particular type of therapeutic encounter or promotes a particular style of consultation, relationship, etc.	*it is a judged thing in terms of how much you can use this on a day-to-day basis with individual patients. And it's a selective thing that you do use, well, you do use selectively. You use it when you need to, certainly not all the time.*<br><br>(GP Group 3)
Placebogenic practice acceptability depends on the belief system of the doctor and the patient	Participants speak of there needing to be agreement between the individual belief system of both the doctor and the patient for acceptability.	*This is a very different scenario to what we've had before where we've had someone who has effectively had no thoughts about something whatsoever and you can then say, 'This has really got every chance of working.' We are in a very different position. This is someone who has done their research; their belief system is such this isn't going to work. Well, you can't suddenly impose your belief system on their belief system, it doesn't work that way and if it did work that way actually, you know, the patient becomes very dependent and there's all sorts of stuff around that that you don't want. So, for me, this is about negotiating some form of change and when you go into the negotiating progress or process, you have to be aware that, actually, should I not succeed, I am going to have to go down one of the alternative avenues. I may not think they're as good but it's better that the patient takes a lesser treatment and takes it, rather than takes what is considered, in your opinion, to be the better treatment, with a sort of 'no-cebo' effect from the patient's perception; so, they're saying, 'Oh, I don't know if I like this, it's not going to work as well.' or they simply might take it*

		entirely against you and go and see a different doctor. So, I think it really is a very high risk strategy.<br><br>*(GP Group 2)*
Placebogenic practice is acceptable if it is not labelled as 'placebo'	Participants discuss that there is something about the label of placebo which governs acceptability.	*Well, I'm not particularly uncomfortable about that because, going back to the point about anti-depressants, I feel I am doing that every time I prescribe an anti-depressant for people with mild to moderate depression because I have a feeling that a lot of what gets better for the patient is either the rest of their psychological therapy, the time, the rearrangement of whatever social difficulties they happen to be in or whatever it is and probably a bit of placebo effect on the anti-depressant whereas an active – I don't think there's any active bit in the pill that's making a difference -*<br><br>(GP Group 2)
Placebogenic practice is not a placebo	Participants disagree that a particular scenario is an example of a placebo.	[*M1]: When you are talking about placebos, are we talking generally sugar pills, I mean that's what we tend to call them. I mean who's to say that an extra boost of sugar is not the kind of treatment that you want. And that it actually does make you better. It might trigger extra endorphins which might make you feel better.*<br><br>(Patient Group 4)
Placebogenic practice is undervalued in the art of medical practice	Discussion about the role of placebogenic practice as a skill in medical practice.	*Actually, I think we underutilise the placebo effect, because I think we could do more with it. So now I think it's a bit of a shame because, in fact, placebos do have some side-effects but, on the whole, less than many of the other tablets*

		we give people. … So I think there's a lost art, almost, that we're not utilising. The problem is it's being seen as deceptive to actually specifically give them a placebo that we don't believe has had any trial behind it to help in their instance. And I think that's where the problem comes about is it's actually our belief, whether it's true or not, or how it comes about for them. And so I suppose, for me, this is a grey area…So I suppose because medicine is so big now, we can't know everything about all the things that we prescribe. We are relying so heavily on other people's information…. So I would say there's a maybe there, and there's certainly times where I suppose I've prescribed antibiotics because the patient won't leave my consulting room so… but I'm sure it's not going to work. In fact, I'm sure I'm not treating a valid infection for them, but they've already spent 25 minutes arguing their case, so I'm going to give it to them. And it's a placebo, and I believe it's a placebo and they believe it's going to work which is the definition of a placebo, so yeah, I suppose, again, it's a vague, grey area. I'm not so hard and fast as everyone else.<br><br>*(GP Group 3)*
Placebogenic practice it is hard to decide	Participants cannot decide whether a placebogenic practice is acceptable or unacceptable. They can list advantages as well as disadvantages close together and find it difficult to come down on one side or the other.	*But I think you have got to be so careful who you target that at, so careful, because the breach of trust and that feeling of breach of trust, can have worse effects I think than the positive effect, if you see what I mean, so it is a balancing act.*

	There can be evidence of dilemmatic thinking and/or indecision.	(Patient group 1)
