# Supplementary material for: Placebos in primary care? a nominal group study explicating UK GP and patient views of six theoretically plausible models of placebo practice
Source: BMJ Open. 2020 Feb 18;10(2):e032524. doi: 10.1136/bmjopen-2019-032524 (PMC7044897; doi:10.1136/bmjopen-2019-032524)
Supplement: Author's manuscript [file bmjopen-2019-032524.draft_revisions.pdf]

BMJ Open is committed to open peer review. As part of this commitment we make the peer review history of every article we publish publicly available.

When an article is published we post the peer reviewers' comments and the authors' responses online. We also post the versions of the paper that were used during peer review. These are the versions that the peer review comments apply to.

The versions of the paper that follow are the versions that were submitted during the peer review process. They are not the versions of record or the final published versions. They should not be cited or distributed as the published version of this manuscript.

BMJ Open is an open access journal and the full, final, typeset and author-corrected version of record of the manuscript is available on our site with no access controls, subscription charges or pay-per-view fees (http://bmjopen.bmj.com).

If you have any questions on BMJ Open's open peer review process please email info.bmjopen@bmj.com

BMJ Open

Placebos in Primary Care? A Nominal Group Study Explicating GP and Patient Views

Journal:	*BMJ Open*
Manuscript ID	bmjopen-2019-032524
Article Type:	Original research
Date Submitted by the Author:	21-Jun-2019

Complete List of Authors:	Ratnapalan, Mohana; University of Southampton, Primary Care and Population Sciences<br>Coghlan, Beverly; ACT Works Ltd<br>Tan, Mengxin; London School of Hygiene and Tropical Medicine, Centre of Global Mental Health; Institute of Psychiatry Psychology and Neuroscience<br>Everitt, Hazel; University of Southampton, Primary Care and Population Sciences, Faculty of Medicine<br>Geraghty, Adam; University of Southampton, Primary Care and Population Sciences<br>Little, Paul; University of Southampton, Primary Care and Population Science;<br>Lewith, George; University of Southampton, Primary Care and Population Sciences<br>Bishop, Felicity; University of Southampton, Centre for Clinical and Community Applications of Health Psychology; University of Southampton, Primary Care and Population Sciences

Keywords:	PRIMARY CARE, QUALITATIVE RESEARCH, placebos, placebo effects, general practice

SCHOLARONE™ Manuscripts

I, the Submitting Author has the right to grant and does grant on behalf of all authors of the Work (as defined in the below author licence), an exclusive licence and/or a non-exclusive licence for contributions from authors who are: i) UK Crown employees; ii) where BMJ has agreed a CC-BY licence shall apply, and/or iii) in accordance with the terms applicable for US Federal Government officers or employees acting as part of their official duties; on a worldwide, perpetual, irrevocable, royalty-free basis to BMJ Publishing Group Ltd ("BMJ") its licensees and where the relevant Journal is co-owned by BMJ to the co-owners of the Journal, to publish the Work in this journal and any other BMJ products and to exploit all rights, as set out in our licence.

The Submitting Author accepts and understands that any supply made under these terms is made by BMJ to the Submitting Author unless you are acting as an employee on behalf of your employer or a postgraduate student of an affiliated institution which is paying any applicable article publishing charge ("APC") for Open Access articles. Where the Submitting Author wishes to make the Work available on an Open Access basis (and intends to pay the relevant APC), the terms of reuse of such Open Access shall be governed by a Creative Commons licence – details of these licences and which Creative Commons licence will apply to this Work are set out in our licence referred to above.

Other than as permitted in any relevant BMJ Author's Self Archiving Policies, I confirm this Work has not been accepted for publication elsewhere, is not being considered for publication elsewhere and does not duplicate material already published. I confirm all authors consent to publication of this Work and authorise the granting of this licence.

For peer review only

Full Title: Placebos in Primary Care? A Nominal Group Study Explicating GP and Patient Views

Authors: Mohana Ratnapalan[1], Beverly Coghlan[2], Mengxin Tan[3,4], Hazel Everitt[1], Adam W A Geraghty[1], Paul Little[1], George Lewith[1], Felicity L Bishop[1,5]

Affiliation:

1. Primary Care and Population Sciences, University of Southampton, Aldermoor Health Centre, Aldermoor Close, Southampton, SO16 5ST
2. ACTWorks Ltd 31b North Street, Emsworth, PO18 8UF
3. London School Hygiene and Tropical Medicine, Centre of Global Mental Health, Keppel Street, Bloomsbury, London, WC1E 7HT
4. Institute of Psychology, Psychiatry and Neuroscience, King's College London 6 De Crespigny Park, Camberwell, London SE5 8AB, UK
5. Centre for Clinical and Community Applications of Health Psychology, Faculty of Environmental and Life Sciences, Building 44 Highfield Campus, University of Southampton, Southampton SO17 1BJ

Corresponding author: Dr Felicity Bishop
Centre for Clinical and Community Applications of Health Psychology, Faculty of Environmental and Life Sciences, Building 44 Highfield Campus, University of Southampton, Southampton SO17 1BJ
Phone: +44 (0)23 8059 9020 Email: F.L.Bishop@southampton.ac.uk

Declarations:

Guarantor: FB

Competing interests: none

Funding: The project "Creating a Taxonomy to Harness the Placebo effect in UK primary care" was funded by the National Institute of Health Research (NIHR) School for Primary Care Research (SPCR) (project number 161). Additional funding for BC was provided by Solent NHS Trust. MR also received funding for part of her research time from the SPCR.

Disclaimer: This paper presents independent research funded by the National Institute of Health Research (NIHR). The views expressed are those of the author(s) and not necessarily those of the NHS, the NIHR, HEE or the Department of Health. The funders had no role in design and conduct of the study; collection, management, analysis, and interpretation of the data; preparation, review, or approval of the manuscript; and decision to submit the manuscript for publication.

Ethical approval: Ethical approval for this study was obtained from the Faculty of Medicine Ethics Committee, University of Southampton (reference number: 4741). Participants gave their informed consent before taking part.

Data sharing: Qualitative coding data available in appendix 1. Further quotations are available, subject to ethical approval, by contacting the authors.

Patient consent for publication: Not required

Author Contributions: FB, GL, PL, BC, HE and AG conceived and designed the study. BC conducted the nominal group meetings. FB, BC, HE, MT and MR were involved in the data analysis and interpretation. MR led the integrated analysis and drafting of the manuscript. All authors contributed to the drafting of the manuscript and take responsibility of the integrity of the data and the analysis.

Acknowledgements: We would like to thank all our participants who gave their time to partake in our nominal groups. We would also like to thank the Primary Care Research Network.

For peer review only - http://bmjopen.bmj.com/site/about/guidelines.xhtml

Abstract

Objectives: To better understand which theoretically plausible placebogenic techniques might be acceptable in UK primary care.

Design: A qualitative study using nominal group technique and thematic analysis. Participants took part in audio-recorded face-to-face nominal groups in which the researcher presented 6 scenarios describing the application in primary care of theoretically plausible placebogenic techniques: (1) Withholding side-effects; (2) Monitoring (3) GP endorsement (4) Idealised consultation (5) Deceptive placebo pills (6) Open-label placebo pills. Participants voted on whether they thought each scenario was acceptable in practice and discussed their reasoning. Votes were tallied and discussions transcribed verbatim.

Setting: Primary care in England.

Participants: 21 GPs in 4 nominal groups and 20 "expert" patients in 5 nominal groups.

Results: Participants found it hard to decide which practices were acceptable and spoke about needing to weigh potential symptomatic benefits against the potential harms of lost trust eroding the therapeutic relationship. Primary care patients and doctors felt it was acceptable to harness placebo effects in practice by patient self-monitoring (scenario 2), by the GP expressing a strongly positive belief in a therapy (3), and by conducting patient-centered, empathic consultations (4). Deceptive placebogenic practices (scenarios 1 and 5) were unacceptable to most groups. Patient and GP groups expressed a diverse range of opinions about open label placebo pills.

Conclusions: Attempts to harness placebo effects in UK primary care are more likely to be accepted and implemented if they focus on enhancing positive patient-centered communication and empathic relationships. Using placebos deceptively is likely to be unacceptable to both GPs and patients. Open-label placebos also do not have clear support; they might be acceptable to some doctors and patients in very limited circumstances - but further evidence, clear information and guidance would be needed.

Keywords: placebos, placebo effects, primary care, general practice, qualitative research

Article Summary: Strengths and limitations of this study

- Nominal group technique and thematic analysis was used to identify key opinions from both GPs and patients on theoretically plausible placebogenic techniques Participants were recruited through research networks and patient charities and sampled to achieve a broad range of views
- Scenarios discussed were carefully constructed to reflect potential placebogenic practice based on clinical and experimental research evidence
- Some nominal groups were small due to the availability of participants.
- It was not always possible to achieve a clear majority opinion on the scenarios

For peer review only

For peer review only - http://bmjopen.bmj.com/site/about/guidelines.xhtml

Manuscript (Word count 3660/4000)

Background

Placebos have an uncertain role in everyday medical practice. They have a long history [1-3] and evidence suggests therapeutic benefit [4-10]. However, there is no broad consensus on how to define placebos nor the ethics of use in clinical practice [11, 12]. Definitions vary between placebos as a substance, a process (e.g. practitioner empathy) or both [11]. This paper defines placebos as substances or processes other than the active ingredients of treatment, which can have substantial effect on symptoms. We define placebo effects as beneficial symptomatic changes triggered by the meaning a person experiences in a healthcare setting [13, 14].

In the UK, there are over 300 million primary care consultations annually [15] with rising demand in the last decade [16]. Within this context, it becomes important to optimise doctor-patient encounters for maximum health benefit. Placebogenic practices, i.e. techniques that can trigger placebo responses in clinical settings, could support cost-effective healthcare, which minimises patient harm from drug side effects and/or enhances the effects of prescribed evidence-based therapies. A recent meta-analysis describes frequent use of placebos in primary care with particularly high use of non-specific therapies (E.g. physician as placebo to exert positive psychological effect) [17]. A meta-ethnographic review of patient and doctor views on placebo practice found acceptable use to patients include therapeutic benefit and giving hope; with health care professionals also citing therapeutic benefit and placebos as clinical management tools [11]. However, few studies directly compared doctors' and patients' views. A meta-analysis on open-label placebos, where patients are honestly informed they are being given placebos found positive clinical effects [18]. However, few qualitative studies have explored patients' or doctors' perspectives on open-label placebos [19]. We used nominal group technique [20-22], a qualitative consensus building technique, to explore and compare how patients and doctors conceive a range of placebogenic practices and why certain practices are more acceptable.

Methods

We gained ethical approval from the Faculty of Medicine Ethics Committee, University of Southampton (reference number: 4741). We did not specifically involve patients or the public in the design, conduct or reporting of this study. However, this study aimed to capture patient views.

Our research team consists of health psychologists (FB, AG), GPs (PL, GL, HE), a psychotherapist (BC), a psychology student (MT) and a GP Academic Clinical Fellow (MR). This range of backgrounds enriched our qualitative analysis, enabling us to bring diverse perspectives to the data and ensuring we explored multiple potential themes, remained open to new ways of conceptualising the data, worked to evidence our interpretations in the raw data, and avoided an idiosyncratic interpretation. Ultimately our approach to data collection and analysis was driven by our pragmatic aim to examine which placebogenic practices might be more or less acceptable to patients and GPs and why.

For peer review only - http://bmjopen.bmj.com/site/about/guidelines.xhtml

Participant Recruitment

We recruited English-speaking GPs and "expert" adult patients, i.e. those with recent experience of using health services and involvement in patient organizations or medical research. We advertised to GPs through the south-west primary care research network and to patients through UK-wide patient associations and health charities (e.g. Pain UK, CFS/ME groups). Individuals expressing interest were mailed a participant information sheet and completed a consent form before participation. We deliberately sought to include GPs and patients of a range of ages and genders and patients with a range of health conditions.

Participants who agreed to participate did so on pre-specified days. The number of people willing to participate determined group size and composition.

Nominal Groups

We structured nominal group meetings as per methodological guidance [20, 22](**Error! Not a valid bookmark self-reference.**). BC, an accredited psychotherapist experienced in facilitation, led the group meetings held in suitable venues (e.g. meeting rooms in GP practices) in 2013.

Table 1: Nominal Group Meeting Structure

Phase	Activity
**Informed Consent**	Facilitator (BC) talks through participant information sheets and consent forms and answers any questions. Participants sign consent forms.
**1: Introduction**	Facilitator introduces the topic, explains our interest in it and asks participants to introduce themselves
**2: Silent reflection**	Participants read the scenario and write comments
**3: Round robin**	Facilitator elicits one comment from each participant and writes this on a flipchart. Discussion not allowed. Continues until comments exhausted.
**4: Discussion**	Facilitator guides discussion of comments on each scenario in turn, using open-ended questions and ensuring all participants had the opportunity to contribute their perspective.
**5: Voting**	Participants vote whether the scenario is acceptable or not. (Undecided was also permitted)
**6: Repeat**	Processes 2 to 5 are repeated in turn for each scenario
**7: Break**	Facilitator counts votes
**8: Discussion**	Results of votes presented and discussed. Each scenario without clear majority is discussed in turn.
**9: Voting**	Second round of voting if no clear majority in first round of voting.
**10: Conclusion**	Results of vote. Facilitator explains future plans and thanks participants.

Nominal groups were presented 6 scenarios for voting and discussion. These scenarios were written by the research team using a taxonomy of five domains of placebogenic techniques [23] derived from experimental and clinical studies [24] to create 6 theoretically plausible placebogenic scenarios for primary care (**Error! Not a valid bookmark self-reference.**). Techniques from the five domains [23] were used to create the scenarios: 1) the patient's beliefs and characteristics informed 'Withholding Side-Effects'; 2) The healthcare setting informed 'Monitoring'; 3) The practitioner's beliefs and characteristics informed 'GP endorsement'; 4)the patient-practitioner interaction informed

'Idealised consultation'. 5) Treatment characteristics informed 'Deceptive'/'Open-label Placebo Pills'. GP groups read scenarios written from the GP perspective.

Table 2: Scenarios for Patient Groups

Scenario	Aspect that might enhance placebo responding
**Scenario 1: "Withholding Side-Effects"** You visit your GP because you have noticed new or worsened symptoms. Your GP examines you, asks you about your symptoms, gives you a diagnosis and decides to prescribe medication. Your GP knows that if she provides you with positive information about the medication you are more likely to notice a benefit. So to make you feel hopeful about your treatment she tells you, truthfully, that research has shown that the majority of patients taking this medication notice a big improvement in their symptoms, and that you too, should notice a big improvement. The medication might have side-effects, but your GP does not tell you about these. This is because she knows that if she *does* tell you about the possible side-effects then you will be more likely to suffer from them.	Giving a positive message may enhance patients' response expectancy; withholding information about medication side effects may reduce the chances of the patient developing them via nocebo mechanisms.
**Scenario 2: "Monitoring''** You visit your GP because you have noticed new or worsened symptoms. Your GP advises you to continue with your usual treatment but requests that you attend the surgery more frequently for on-going review and monitoring of your condition. She also asks you to monitor your symptoms on a daily basis and report back to her at your next visit. She provides you with a special symptom-monitoring diary to help you to do this.	The use of regular monitoring and review may increase awareness of symptom changes and potentially motivate behavioural changes. .
**Scenario 3: "GP endorsement''** You visit your GP because you have noticed new or worsened symptoms. Your GP examines you, asks you about your symptoms, gives you a diagnosis and offers to prescribe a particular medication. You have heard of this medication and are not sure how effective it will be and ask if there are any other treatments you could try instead. Your GP says that there are but that he strongly believes (based on his experience with other patients and from published research) that the medication he wants to prescribe provides absolutely the best chance of reducing your symptoms in the shortest time.	Conveying the clinicians' strong personal beliefs about a particular medication may enhance patients' response expectancy.
**Scenario 4: "Idealised consultation''** You visit your GP because you have noticed new or worsened symptoms. Your appointment is with the same GP you always see. He greets you warmly and seems pleased to see you. He turns away from his computer screen and gives you his full attention. He is very interested and concerned about what you tell him. He asks you many detailed questions about how the symptoms started and how they are now affecting your daily life. He thoroughly examines you. He genuinely seems to be interested in you as a person and not as just a collection of symptoms. He allows you time to ask questions and even though he does not know all of the answers he provides as much information as he can and says he will try to find out more and will get back to you later in the day by telephone. He tells you that he would prefer it if you continue to make your appointments with him in future.	Enhanced attention, more time, warm and empathic and collaborative style may enhance perception of empathy, validation, and response expectancy.
**Scenario 5: "Deceptive placebo pills''** You attend your GP surgery because you have noticed new or worsened symptoms. Your GP examines you, asks you about your symptoms, gives you a diagnosis and recommends a prescription for medication. She tells you that	Prescribing a placebo medication deceptively may enhance response expectancy and

For peer review only - http://bmjopen.bmj.com/site/about/guidelines.xhtml

research has shown that by taking this medication three times a day for at least a week, your symptoms will get better. What she **does not** tell you is that the medication she will be prescribing is actually a "placebo" pill that contains **no real medicine.**	engender a conditioned response to pill taking.
**Scenario 6: " Open-label placebo pills''** You attend your GP surgery because you have noticed new or worsened symptoms. Your GP examines you, asks you about your symptoms, gives you a diagnosis and recommends a prescription for medication. She tells you that research has shown that by taking this medication three times a day for at least a week, your symptoms will get better. What she **does** tell you is that the medication she will be prescribing is actually a "placebo" pill that contains **no real medicine.**	Prescribing a placebo medication openly may enhance response expectancy and engender a conditioned response to pill taking.

Data Analysis

Each meeting was audio-recorded, transcribed verbatim and anonymized.

Our analytical approach rested on principles described by McMillian et al and encompassed attending to both participants' votes and qualitative discussions [21]. Votes were counted and each group was classified according to whether the majority of participants deemed each scenario acceptable, unacceptable, or 'no clear majority'. To analyse the discussions we used thematic analysis [25] with constant comparison between groups and scenarios. After repeated reading of transcripts, initial low-level inductive codes were developed independently for the GP and patient transcripts by MR and MT respectively, using Nvivo 12 to facilitate coding and maintain an audit trail. Low level codes were reviewed by FB, MR, and MT who iteratively developed higher level codes by merging similar low-level codes and combining them into a hierarchical structure. MR led the search for themes by comparing and contrasting codes across scenarios and across GP and patient groups, reviewing potential themes for fit with the raw data. MR, HE, and FB discussed which themes best captured GPs' and patients' reasoning around placebogenic practice and agreed on the final 16 themes (see Appendix 1). MR then integrated the qualitative themes with the vote frequency data using an iterative process comparing votes and themes (a) within groups across individual scenarios and (b) within scenarios across groups. This analysis was developed and agreed by all authors and is presented below. We used the SRQR checklist when writing our report[26].

Results

Participant characteristics

21 GPs and 20 patients (**Error! Not a valid bookmark self-reference.**) participated in 9 nominal groups (4 GP and 5 patient groups); with 2 to 8 participants and lasting 75-100 minutes per group. Most GPs (n=17, 81%) were working full-time. 2 patients completed sixth form or college, 4 university undergraduate, and 9 post-graduate (5 did not disclose). 15 patients disclosed their general health status as follows: very good, n=7 (35%), good 1 (5%), fair 6 (30%), bad 1 (5%). Patients' self-reported health conditions included: chronic pain, irritable bowel syndrome, cancers and diabetes.

For peer review only - http://bmjopen.bmj.com/site/about/guidelines.xhtml

Table 3: Demographics

	GP	Patient
**Total n**	21	20
**Number of males (%)**	12 (57%)	7 (35%)
**Number of females (%)**	9 (43%)	13 (65%)
**Mean age (SD) ****	42 (9.2)	56.3 (12.7)
**Mean years GP (SD)*****	15 (10.1)	-
**Range of group size (mean)**	3-8 (5)	2 – 7 (4)

Notes: ** 5 not disclosed; * 3 not disclosed. Undisclosed demographic data comes from different nominal groups and is not isolated missing data for any single group.**

Qualitative Analysis

Overview

Participants found it hard to decide whether each placebogenic practice was acceptable. Patients and GPs spoke about the tension between balancing positive effects of placebogenic practice against harmful erosion of the therapeutic relationship from loss of trust.

''But I think you have got to be so careful … because the breach of trust and that feeling of breach of trust, can have worse effects I think than the positive effect… so it is a balancing act.''

(Patient Group 1)

''… the nice thing about GPs is having the ongoing patient relationship. So we' re also trying to build a relationship and that's, obviously, part of a placebo effect. But if you tell patients it's going to work brilliantly and it doesn't then that slightly damages their trust, versus if you tell them that they might get a side-effect but it will settle down… But again, it's either damaging or enhancing in the GP relationship, as well.''

(GP Group 3)

Despite these tensions, there were some consistent patterns in the voting (**Error! Not a valid bookmark self-reference.**). 'Monitoring' and 'GP endorsement' were acceptable to all GP groups while the 'Idealised consultation' was acceptable to all GP and patient groups. The arguments that participants offered in the discussions to justify their votes are explored below.

Table 4: Tabulated group level voting on acceptability of 6 scenarios of placebogenic practice

Scenario	Acceptable	No clear majority	Unacceptable
1. "Withholding side effects"	Δ Δ	Δ Δ ◯	Δ ◯◯◯
2. "Monitoring"	Δ Δ Δ Δ ◯ ◯◯◯	Δ	
3. "GP endorsement"	Δ Δ Δ Δ ◯◯◯ ◯	Δ	

For peer review only - http://bmjopen.bmj.com/site/about/guidelines.xhtml

4. "Idealised consultation"	Δ Δ Δ Δ Δ ◯◯◯ ◯		
5. "Deceptive placebo pills"		Δ	Δ Δ Δ Δ ◯◯ ◯◯
6. "Open-label placebo pills"	◯◯	Δ Δ Δ ◯	Δ Δ ◯

◯ = GP groups (n=4) Δ = Patient groups (n=5)

GPs and patients felt that "Monitoring" empowered patients by providing patient centred care. GPs argued that involving the patient and using time as diagnostic tool could help them consult more effectively, but expressed concern that over-emphasising symptoms could lead to psychological harms (e.g. generating anxiety). The acceptability of this scenario was felt to depend on the medical condition, the patient's characteristics (e.g. age), the work required of the patient for self-monitoring, the disease process and the symptom severity.

''..it would also provide me with more control …over my condition… by being aware of change.''
(Patient Group 5)

''Potentially, I'll say, I would do this – if, in the 10 minutes I've got available, I really haven't got a true reflection of you know, what the symptom pattern is and what the effect on the patient really is, then it's just a way of extending the consultation over a period of time and to actually gather that information.''
(GP Group 2)

GP groups discussed how the GPs' experience and the evidence-base would influence the acceptability of "GP endorsement". GPs felt there needed to be a published evidence-based benefit or personal experience of likely therapeutic benefit to endorse a therapy. Patients felt that "GP endorsement" might be more acceptable in the context of more egalitarian doctor-patient relationships.

''Again I think it depends on the relationship I as the patient had with that GP, whether it was a relationship I felt was equal, or not. If it was then I would be more inclined to go along with that advice. If I felt it was more of a paternalistic relationship than I would be questioning why, why does he think this is the best one. I'd want more information about that drug, and also to discuss whatever it is that I've heard about this medication and why I've heard it's not necessarily the best thing. And also to be sure that it's not being prescribed because it's the latest drug that pharmaceuticals are pushing and this is a really good one and it will do all singing, all dancing.''
(Patient Group 2)

The continuity of care within the "Idealised consultation" was particularly well-received. GPs felt continuity of care enhanced their job satisfaction and improved their understanding the psychosocial context of their patients by permitting long-term relationships to develop with patients and families. GPs felt continuity provided a directed trajectory of care that disjointed multi-

For peer review only

practitioner led care might not provide. Patients agreed and valued the idea of seeing a practitioner who knew their story.

Despite universal acceptance of the "Idealised consultation", GPs and patients also expressed concerns about this. GPs were concerned that knowing their patients too well could lead to harm from cognitive bias and encourage patients to become overly reliant on one doctor and subsequently come to harm from delaying presenting if that doctor was unavailable. GPs and patients both expressed concerns that this scenario was unrealistic given workloads and/or would increase GP workload, which may in turn negatively affect care, and that this type of practice could blur doctor-patient boundaries.

''And sometimes you know your patient so well that you just don't see that they're losing weight or they're becoming hypothyroid or something.''

(GP Group 4)

'' [F1]: Well, I say a GP from heaven! …I would have full trust and confidence in the GP, if ever they had that sort of response to you and a welcoming aspect to it, and naturally being eye-contact, focussing with you, and receptive both ways. And interested in you and there is communication, as a key factor. And to be able to leave that surgery knowing that you have got some form of support out there, in such an isolating situation whenever you are in chronic pain.

[F3]: I mean I think even the admission that he doesn't know all the answers is reassuring, because GPs are what GPs are, they are not specialists, they have to know something about a lot of things, but not necessarily deep down into one specialisation, but they know where to go….''

(Patient Group 1)

Deception in Placebogenic Practice

"Withholding side-effects" and "Deceptive placebo pills" both involve deception. Most groups found "Withholding side effects" unacceptable or impossible to reach a majority judgement, while all but one group found "Deceptive placebo pills" unacceptable.

GPs and patients were worried about the risks of physical and psychological harm and damage to the GP-patient relationship from withholding information about side- effects. For example, one GP group was concerned about patient harm from an accident if they were unaware of potential impaired function. One patient group discussed how an unexpected side-effect might cause anxiety that this was a new health problem. Patients felt that withholding information disempowers them and being inconsistent with patient-centred care where ultimate autonomy rests with the patient to make informed decisions. GP groups also discussed medico-legal and policy issues. They worried about medico-legal implications of non-disclosure and discussed how government targets may alter their discussions about medication.

For peer review only - http://bmjopen.bmj.com/site/about/guidelines.xhtml

For peer review only

''Yes, I think my views change with time, too, and the outside world we're working in. I'm far more likely to give somebody an ACE inhibitor now than I was five years ago, simply because its part of the QOF targets. And that has a big effect on the way I will sell an ACE, give an ACE and encourage the patient to use it. I probably use quite a lot of the 'Doctor knows best' concept in the consultation to push that particular drug. Because there is a monetary target involved with it.''

(GP Group 3)

''To me, it depends upon the frequency and the severity of the side effects. Because if they're rare and minor I would be completely comfortable with it, if they're serious or very frequent I'd be uncomfortable with it because you risk loss of trust…. And again, there's the risks, especially if … it impaired their function and they had an accident or, you know, there's risks to not telling people about potential side effects.''

(GP Group 1)

''Yes. If you're expecting a patient to take a drug then they have to understand potentially what the problems or issues could be. I mean, you know, even if they are fairly minor I think most people understand that it's only a 1% chance of things happening, but at least it's their decision to take that drug or to take that treatment, and they can't take that decision if they're being pushed, if you like, being pushed or being persuaded to do it if they don't get the full information and it's not the doctor's decision, it's the patient's decision.''

(Patient Group 4)

In contrast, "Deceptive placebo pills" involved more active deception and was felt to be dishonest. GPs were concerned that this was ethically unacceptable practice and this drove their decision-making irrespective of potential benefit from placebo pills. GPs felt that it was imperative that patients were able to make fully informed decisions about therapy in this scenario. There was fear of repercussions from the General Medical Council, as the use of placebo pills is not, currently, incorporated into professional codes of practice nor accepted within the wider cultural context of medical practice. One GP group felt there might be a role for deceptive placebo pills in (unspecified) mental illness. However, the same group expressed a tension between personal ethics and accepted codes or standards of practice.

Similarly, patients expressed discomfort with receiving unknown substances and judged the deceit involved as ethically unacceptable. Patients spoke of risk of psychological harm from feeling that their symptoms were not 'real' and were "all in the mind", with some seeing placebo pills as not real therapy for real symptoms. They also worried that placebo pills would be a way for GPs to avoid properly investigating their problems. Patients spoke of subverting the placebogenic effect by seeking information about the pills outside the consultation (e.g. online). Both patients and GPs expressed concerns about deceptive prescribing eroding the doctor-patient relationship.

''[M1]: Do you believe in a placebo?

[F1]: …. I do believe in it but that's not actually what's being asked…I believe that the patient should know what it is they're signing up to. So I'm really happy in a proper clinical trial where you're told you could go into the placebo arm or you could go into the arm where you will get the drug. That's absolutely fully acceptable and you then don't know whether you've got the placebo. That's great, but this is wrong, this is underhand.

For peer review only - http://bmjopen.bmj.com/site/about/guidelines.xhtml

[F2]: I think it's the GP judging you, thinking he or she knows you really well. I mean how well do they know you from a 5-10 minute consultation. You know, have they asked you about other things going on in your life? Other issues and things, or are they just focussing on that one aspect.

[F1]: Yeah, give them a dummy pill and then they'll go away and be quiet. As opposed to actually, you know, getting down to what the issue actually is.''

(Patient Group 4)

''And how do you feel about that? How do you defend against that? And what is the patient going to think of you next time they see you, if they realise it was a fake pill, so to speak? And how do they have confidence in you from thereon in?''

(GP Group 2)

''I could live with it ethically, I think the problem is the GMC code of practice, isn't it?''

(GP Group 1)

Open-label placebo pills

In contrast to "Deceptive placebo pills", "Open-label placebo pills" removed the element of deception with placebo pills. Despite many of the objections to "Deceptive placebo pills" focusing on the deception per se, removing the deceptive element did not lead to a complete shift towards groups seeing placebos as acceptable. Patients felt that the acceptability of open-label placebo depended on the medical condition and their trust in the GP. Patients were not happy to pay a prescription charge for what they saw as an inert pill and if they saw placebo as inappropriate or ineffective they argued that this would weaken the therapeutic effect.

''That it was a placebo and that it was found to work in other people, I'd think great, I'll give it a go, yeah. I'd be quite happy about that, it's the not being told that I have the problem with.''

(Patient Group 2)

''Then I'd want my £7 whatever it is prescription. Give me a bag of sugar instead.''

(Patient Group 4)

Some GPs felt that prescribing alone was not enough and additional positive talk and a cultural shift would be required. Others worried that patients would stop seeing them if they prescribed placebo pills.

*''**M1** … I've never personally done that but I know when I was doing paediatrics there was a child with quite profound functional symptoms …and they knew they were having oral saline and they got better, they improved. So I would be more comfortable with that but I've never had a clinical context where I've had the courage to do it but if it …*

***F2** Me neither.*

For peer review only - http://bmjopen.bmj.com/site/about/guidelines.xhtml

***M1** But if it was more of a sort of cultural thing I would be very glad we're doing that.''*

(GP Group 4)

''I just think it's mad. If I did that with my patients they'd never come and see me again and say, "I'll get a doctor that gives me actual medicine."

(GP Group 1)

Discussion

Our study captures both GP and patient views and offers new insights on the real-world application of placebos. We found that placebogenic practice with deception is very clearly not acceptable and for open-label placebo pills, there was no clear judgement of acceptability from any of the patient groups. This extends on previous studies, which suggest that GPs found deceptive placebogenic practice unacceptable [27, 28] and some patients feel it is important for any placebogenic practice to respect patient autonomy [29]. By focusing on theoretically plausible placebogenic scenarios, we provide new insights to placebogenic practices with potential for implementation to enhance patient outcomes in clinical practice and clarified the psychological and sociocultural barriers that would need to be overcome.

We found the acceptability of placebogenic practice is difficult to determine and even 'acceptable' scenarios elicited talk of caveats, as did a recent meta-ethnography [11]. Caveats to acceptability identified in our study include, but are not limited to, considerations of: medical condition; individual patient; individual doctor; regulatory norms; government prescribing targets; GMC guidance and what is viewed as acceptable practice to other medical colleagues (i.e., social norms). This suggests that any generic guidelines proclaiming a type of practice as either 'acceptable' or 'unacceptable' may not capture the views of GPs and patients as key stakeholders and may be problematic. It may be more useful to develop guidance that highlights important considerations and contexts for placebogenic practice.

Our study is limited by non-blinded voting. The group method means discussions must be interpreted in their social and cultural context, and not as individuals' personal beliefs. It is informative, although not surprising, that the GP groups discussed clinical practice norms while the patient groups (comprising expert patients who were typically accustomed to acting as patient advocates) drew heavily on the notion of patient autonomy. Despite attempts to purposively sample a diverse group, some nominal groups were small with 2 or 3 participants. Findings indicate patterns of views held by our participants, all of whom volunteered their time and so might be more interested in and/or hold stronger views about placebo effects than non-participants.

Conclusions and Future Work

Our study helps inform future work on placebogenic practice and provides clinicians with improved understanding of what peers and patients would find acceptable, whilst acknowledging this is a complex area with diverse opinions. Our study suggests that open-label placebo pills are not fully

For peer review only - http://bmjopen.bmj.com/site/about/guidelines.xhtml

acceptable and future translational work could consider prescription costs among other issues. Additional research testing acceptable placebogenic techniques in clinical settings is needed to help inform clinicians on the effectiveness of these practices in clinical practice.

For peer review only

For peer review only - http://bmjopen.bmj.com/site/about/guidelines.xhtml

References

1. Booth C. The rod of Aesculapios: John Haygarth (1740-1827) and Perkins' metallic tractors. *Journal of medical biography* 2005; 13: 155-161. 2005/08/02. DOI: 10.1177/096777200501300310.
2. Czerniak E and Davidson M. Placebo, a historical perspective. *European neuropsychopharmacology : the journal of the European College of Neuropsychopharmacology* 2012; 22: 770-774. 2012/05/23. DOI: 10.1016/j.euroneuro.2012.04.003.
3. Jutte R. The early history of the placebo. *Complementary therapies in medicine* 2013; 21: 94-97. 2013/03/19. DOI: 10.1016/j.ctim.2012.06.002.
4. Zhang W, Robertson J, Jones AC, et al. The placebo effect and its determinants in osteoarthritis: meta-analysis of randomised controlled trials. *Annals of the rheumatic diseases* 2008; 67: 1716-1723. 2008/06/11. DOI: 10.1136/ard.2008.092015.
5. Kirsch I, Deacon BJ, Huedo-Medina TB, et al. Initial severity and antidepressant benefits: a meta-analysis of data submitted to the Food and Drug Administration. *PLoS medicine* 2008; 5: e45. 2008/02/29. DOI: 10.1371/journal.pmed.0050045.
6. Patel SM, Stason WB, Legedza A, et al. The placebo effect in irritable bowel syndrome trials: a meta-analysis. *Neurogastroenterology and motility : the official journal of the European Gastrointestinal Motility Society* 2005; 17: 332-340. 2005/05/27. DOI: 10.1111/j.1365-2982.2005.00650.x.
7. Howick J, Friedemann C, Tsakok M, et al. Are treatments more effective than placebos? A systematic review and meta-analysis. *PloS one* 2013; 8: e62599. 2013/05/22. DOI: 10.1371/journal.pone.0062599.
8. Charlesworth JEG, Petkovic G, Kelley JM, et al. Effects of placebos without deception compared with no treatment: A systematic review and meta-analysis. *Journal of evidence-based medicine* 2017; 10: 97-107. 2017/04/30. DOI: 10.1111/jebm.12251.
9. Kaptchuk TJ, Friedlander E, Kelley JM, et al. Placebos without deception: a randomized controlled trial in irritable bowel syndrome. *PloS one* 2010; 5: e15591. 2011/01/05. DOI: 10.1371/journal.pone.0015591.
10. Hrobjartsson A and Gotzsche PC. Placebo interventions for all clinical conditions. *The Cochrane database of systematic reviews* 2010: Cd003974. 2010/01/22. DOI: 10.1002/14651858.CD003974.pub3.
11. Hardman DI, Geraghty AW, Lewith G, et al. From substance to process: A meta-ethnographic review of how healthcare professionals and patients understand placebos and their effects in primary care. *Health (London, England : 1997)* 2018: 1363459318800169. 2018/09/22. DOI: 10.1177/1363459318800169.
12. Linde K, Friedrichs C, Alscher A, et al. The use of placebo and non-specific therapies and their relation to basic professional attitudes and the use of complementary therapies among German physicians--a cross-sectional survey. *PloS one* 2014; 9: e92938. 2014/04/04. DOI: 10.1371/journal.pone.0092938.
13. Brody H. The placebo response. Recent research and implications for family medicine. *The Journal of family practice* 2000; 49: 649-654. 2000/08/03.
14. Moerman DE and Jonas WB. Deconstructing the placebo effect and finding the meaning response. *Annals of internal medicine* 2002; 136: 471-476. 2002/03/20.
15. Digital N. Appointments in General Practice, October 2018 https://digital.nhs.uk/data-and-information/publications/statistical/appointments-in-general-practice/oct-2018 (2018, accessed 08/01/2019 2019).
16. Baird B CA, Honeyman M, Maguire D, Das P. *Understanding pressures in general practice*. May 2016 2016. The King's Fund.

For peer review only - http://bmjopen.bmj.com/site/about/guidelines.xhtml

17. Linde K, Atmann O, Meissner K, et al. How often do general practitioners use placebos and non-specific interventions? Systematic review and meta-analysis of surveys. *PloS one* 2018; 13: e0202211. 2018/08/25. DOI: 10.1371/journal.pone.0202211.
18. Charlesworth JEG, Petkovic G, Kelley JM, et al. Effects of placebos without deception compared with no treatment: A systematic review and meta-analysis. *Journal of evidence-based medicine* 2017; 10: 97-107. DOI: doi:10.1111/jebm.12251.
19. Kaptchuk TJ and Miller FG. Open label placebo: can honestly prescribed placebos evoke meaningful therapeutic benefits? *BMJ (Clinical research ed)* 2018; 363: k3889. DOI: 10.1136/bmj.k3889.
20. Van de Ven AH and Delbecq AL. The effectiveness of nominal, delphi, and interacting group decision making processes. *Academy of Management Journal* 1974; 17: 605-621. DOI: 10.2307/255641.
21. McMillan SS, Kelly F, Sav A, et al. Using the Nominal Group Technique: how to analyse across multiple groups. *Health Services and Outcomes Research Methodology* 2014; 14: 92-108. journal article. DOI: 10.1007/s10742-014-0121-1.
22. McMillan SS, King M and Tully MP. How to use the nominal group and Delphi techniques. *International journal of clinical pharmacy* 2016; 38: 655-662. 2016/02/06. DOI: 10.1007/s11096-016-0257-x.
23. Di Blasi Z, Harkness E, Ernst E, et al. Influence of context effects on health outcomes: a systematic review. *Lancet (London, England)* 2001; 357: 757-762. 2001/03/20.
24. Bishop FL, Coghlan B, Geraghty AW, et al. What techniques might be used to harness placebo effects in non-malignant pain? A literature review and survey to develop a taxonomy. *BMJ open* 2017; 7: e015516. 2017/07/02. DOI: 10.1136/bmjopen-2016-015516.
25. Braun V and Clarke V. Using thematic analysis in psychology. *Qualitative Research in Psychology* 2006; 3: 77-101. DOI: 10.1191/1478088706qp063oa.
26. O'Brien BC, Harris IB, Beckman TJ, et al. Standards for reporting qualitative research: a synthesis of recommendations. *Academic medicine : journal of the Association of American Medical Colleges* 2014; 89: 1245-1251. 2014/07/01. DOI: 10.1097/acm.0000000000000388.
27. Howick J, Bishop FL, Heneghan C, et al. Placebo use in the United kingdom: results from a national survey of primary care practitioners. *PloS one* 2013; 8: e58247. 2013/03/26. DOI: 10.1371/journal.pone.0058247.
28. Bishop FL, Howick J, Heneghan C, et al. Placebo use in the UK: a qualitative study exploring GPs' views on placebo effects in clinical practice. *Family practice* 2014; 31: 357-363. 2014/04/17. DOI: 10.1093/fampra/cmu016.
29. Bishop FL, Aizlewood L and Adams AE. When and why placebo-prescribing is acceptable and unacceptable: a focus group study of patients' views. *PloS one* 2014; 9: e101822. 2014/07/10. DOI: 10.1371/journal.pone.0101822.

For peer review only

For peer review only - http://bmjopen.bmj.com/site/about/guidelines.xhtml

Appendix 1: Summary of Themes

Theme	Definition	Example quote
Consultation and clinical practice behaviours	General talk which is not necessarily specific to placebogenic practice but which dictates the models of consultation and also broader consideration about working life of a GP.	*I think very few medications I prescribe actually have the best chance of reducing anyone's symptoms in a short time. The majority of medications I'm prescribing are preventative, and that's when you often have to do the sales job, because they're not going to have any change in their symptoms but you just try and give them what's needed to treat them, or I have done. So I think the preventative thing is quite interesting. But I would use the technique at times.*<br><br>(GP Group 3)
Patient perspectives on GP behaviour	General talk about the need for GPs to treat patients as individuals and how GPs should respect patients, e.g. by how they listen and talk to them. Not linked to placebogenic practice. Mainly derived from general talk in the patient groups about past experiences with GPs.	*Yes, because I think that all treatment should be a holistic approach, in that you have got to treat the whole person. It is all very well treating one condition, but sometimes when there are multiple conditions, or it is one condition that has multiple effects, you need to look at the actual person, and to me, very much, this was the approach of looking at one person, and making them feel … and they are taking part in it. And I think that is important.*<br><br>(Patient Group 1)
Placebo models	Talk that alludes to how participants think placebos might operate to generate effects on patients. How do they work, if indeed they do work? Not specifically linked to a particular placebogenic practice under discussion,	*Well this is where the placebo effect comes in – the expectation*<br><br>(Patient Group 4)

For peer review only

	rather this is more general talk about the mechanisms thought to possibly underpin placebo effects.	
Placebogenic practice – health system and medico-legal framework consideration	Aspects of the wider health system that are relevant to the acceptability of placebogenic practice e.g. GMC rules or health targets or legal framework of clinical practice	*And again, there's the risks, especially if there's, you know, it impaired their function and they had an accident or, you know, there's risks to not telling people about potential side effects.*<br><br>(GP Group 1)
Placebogenic practice – honesty, ethical practice and disclosure	Talk about the acceptability and ethical issues around honesty, deception, and complete/incomplete disclosure. About the information that "should" be provided to patients, the information that patients want, and why this is seen as important. Talk about the moral imperative for full information and informed consent, as well as occasional situations where these things are deemed non-essential. Includes talk about the circumstances in which some dishonesty or incomplete disclosure might be tolerated.	*That's unethical. So, if you've got a 1 in 10 chance that actually taking this medication is going to cause you some significant harm, we've got to be completely open about this.*<br><br>(GP Group 2)
Placebogenic practice – patient considerations	Aspects of the patient (e.g. characteristics, beliefs, medical condition, clinical history) that are relevant to considering how acceptable a placebogenic practice is. Does NOT include talk about how it is important to treat patients as individuals or to know one's patient very well (this is coded within placebogenic practice - therapeutic encounter considerations).	*The only observation I made just really when we started talking is that patients who may have that suspicious mind-set about drugs anyway tend to research side effect pretty effectively anyway. Again, if you know your patient you kind of reinforce the patient information leaflet about all the bad things that might happen.*<br><br>(GP Group 4)
Placebogenic practice – patient outcomes	The effects that participants think might flow from a particular placebogenic practice. These are effects on the patient as an individual (as	*F1: It's interesting because it could be that some people might think, oh you know, she cares about me and she wants*

For peer review only

	opposed to their behaviour in relation to consulting and the doctor-patient encounter/relationship). Includes potential benefits and harms, of all types (e.g. includes psychological, physical, financial considerations). Also includes talk about the possible lack of effects of a placebogenic practice - i.e. when participants think that it would not work.	*to see me more, that's a positive thing,*<br><br>*F2: But it might also be a sign of added interest from –*<br><br>*F1: Yes, staying on top of it, you know, wanting to be on your shoulders, not fobbed off with a diary.*<br><br>(Patient Group 2)
Placebogenic practice – practitioner consequences	The effects that participants this might stem from a particular placebogenic practice. These are effects on the doctor as an individual and could include potential benefits and harms of all types.	*I suppose I would use it for a few reasons as well. One would be both to better inform myself, because often patients come in with these vague symptoms and you're never quite sure what's actually happening, because they don't almost really know themselves. So actually getting them to sit down and actually write it out sometimes is very helpful, again, for them to establish, actually, this is the pattern of work. Maybe it isn't as bad as I thought it was because, of course, patients will often think the worst. So that can be really helpful. I think I probably also have used it as a procrastination technique every now and again, because I do think a medication will work but it just needs a bit more time.*<br><br>(GP Group 3)
Placebogenic practice – the practitioner considerations	Properties, characteristics, etc. of the practitioner that are relevant to considering the acceptability of a placebogenic practice. Includes discussion related to the practitioner's status, their qualifications and expertise, and their intentions guiding the placebogenic practice.	*Male 3: I'm not certain that last statement or sentence, 'if you tell him about it he'll be more likely to suffer from them' is true. I think there's a small cohort of patients where you tell them the side effects they will get it, and I could name a few patients where if I say you're going to get cough*

For peer review only

		with giving an ace inhibitor, they will cough. So those are the ones where arguably actually I would not tell them that you might get a cough about it. But I think on the whole patients –<br><br>*Male 1: Would you be happy for one of your patients to come back to you nine months later and say, "I've had this cough for nine months," but they weren't aware it was side effect and they've had to live with that for nine months because you hadn't told them about it?*<br><br>*Male 3: I'd happily live with that, it's only a bit of a dry cough, it's not the end of the world, it's not going to kill –*<br><br>(GP Group 1)
Placebogenic practice – therapeutic encounter consequences	Talk about consequences of the placebogenic practice for the therapeutic encounter, the doctor-patient relationship, and future consultations. Includes talk about both positive and negative consequences.	*if they're serious or very frequent I'd be uncomfortable with it because you risk loss of trust, I think, from your patient if you don't tell them.*<br><br>(GP Group 1)
Placebogenic practice – therapeutic encounter considerations	Issues about the doctor-patient relationship in general and the consultation in particular that are deemed relevant to considering the acceptability of a placebogenic practice. Incudes discussion of the therapeutic encounter and its characteristics and how these might influence whether a particular practice is acceptable. Also includes discussion of how a placebogenic practice itself represents a particular type of therapeutic encounter or promotes a particular style of consultation, relationship, etc.	*That I might ask are there implications if I discontinue treatment? You said I think appropriately I might ask for more information, specifically about discontinuing treatment, I mean discontinuing the meds, yeah, treatment, if that were like it is with antibiotics.*<br><br>(Patient Group 2)

For peer review only

Placebogenic practice acceptability depends on the belief system of the doctor and the patient	Participants speak of there needing to be agreement between the individual belief system of both the doctor and the patient for acceptability.	*Yeah, I'm more comfortable with this. It sounds as though the patient's done a bit of background work. They're actually aware of the particular (inaudible 0:57:01) and for some peculiar reason, they say, 'Actually, I'm particularly keen on that one, I'd like to try something else.' and that I know full well, in this scenario, 'Ok, let's just have a chat about what your concerns are.' and unless you're able to specifically nail those concerns, just don't go there and trying to impose your will – well, you're gonna to lose this one. This is a very different scenario to what we've had before where we've had someone who has effectively had no thoughts about something whatsoever and you can then say, 'This has really got every chance of working.' We are in a very different position. This is someone who has done their research; their belief system is such this isn't going to work. Well, you can't suddenly impose your belief system on their belief system, it doesn't work that way and if it did work that way actually, you know, the patient becomes very dependent and there's all sorts of stuff around that that you don't want. So, for me, this is about negotiating some form of change and when you go into the negotiating progress or process, you have to be aware that, actually, should I not succeed, I am going to have to go down one of the alternative avenues. I may not think they're as good but it's better that the patient takes a lesser treatment and takes it, rather than takes*

For peer review only

		what is considered, in your opinion, to be the better treatment, with a sort of 'no-cebo' effect from the patient's perception; so, they're saying, 'Oh, I don't know if I like this, it's not going to work as well.' or they simply might take it entirely against you and go and see a different doctor. So, I think it really is a very high risk strategy.<br><br>*(GP Group 2)*
Placebogenic practice is acceptable if it is not labelled as 'placebo'	Participants discuss that there is something about the label of placebo which governs acceptability.	Well, I'm not particularly uNComfortable about that because, going back to the point about anti-depressants, I feel I am doing that every time I prescribe an anti-depressant for people with mild to moderate depression because I have a feeling that a lot of what gets better for the patient is either the rest of their psychological therapy, the time, the rearrangement of whatever social difficulties they happen to be in or whatever it is and probably a bit of placebo effect on the anti-depressant whereas an active – I don't think there's any active bit in the pill that's making a differeNCe but for this particular patient group then – I think there was a review recently actually - just in a slight diversion – about – it was looking at Tricyclics against active placebos, so placebos that were formulated to exhibit the same kind of side effects and there is even a graph(?) of response where, these are all generations of anti-depressants but it was actually really interesting -<br><br>(GP Group 2)

For peer review only

Placebogenic practice is not a placebo	Participants disagree that a particular scenario is an example of a placebo.	*[M1]: When you are talking about placebos, are we talking generally sugar pills, I mean that's what we tend to call them. I mean who's to say that an extra boost of sugar is not the kind of treatment that you want. And that it actually does make you better. It might trigger extra endorphins which might make you feel better.*<br><br>*[F2]: Chocolate's always good for you.*<br><br>(Patient Group 4)
Placebogenic practice is undervalued in the art of medical practice	Discussion about the role of placebogenic practice as a skill in medical practice.	*Actually, I think we underutilise the placebo effect, because I think we could do more with it. So now I think it's a bit of a shame because, in fact, placebos do have some side-effects but, on the whole, less than many of the other tablets we give people. And the oncology was a quest example of… my oncologist in New Zealand always said, "People who walk in here positive are going to be the ones that are cured. It doesn't really matter whether I treat them with whatever toxic medication I've got." So I think there's a lost art, almost, that we're not utilising. The problem is it's being seen as deceptive to actually specifically give them a placebo that we don't believe has had any trial behind it to help in their instance. And I think that's where the problem comes about is it's actually our belief, whether it's true or not, or how it comes about for them. And so I suppose, for me, this is a grey area. I know, again, vitamin tablets, I know one woman who used to be on*

		paracetamol because it helped her sleep every night. As far as I could tell she was having pain, so it wasn't controlling pain relief. Definitely helped her sleep, though, probably better in the long-term than benzos, but who really knows? But she was happy on it, and whether I prescribed it or whether she went down and bought it at the chemist is a moot point, and we probably kept it on her medication list so we knew we weren't going to poison her with anything else. So I suppose because medicine is so big now, we can't know everything about all the things that we prescribe. We are relying so heavily on other people's information, and I'm not entirely convinced the drug companies necessarily had our best interest at heart, or the patient's best interest at heart, even. I think probably the shareholders' best interests come first and then possibly patient second and certainly GPs lower down the chain. So I would say there's a maybe there, and there's certainly times where I suppose I've prescribed antibiotics because the patient won't leave my consulting room so… but I'm sure it's not going to work. In fact, I'm sure I'm not treating a valid infection for them, but they've already spent 25 minutes arguing their case, so I'm going to give it to them. And it's a placebo, and I believe it's a placebo and they believe it's going to work which is the definition of a placebo, so yeah, I suppose, again, it's a vague, grey area.

For peer review only

		I'm not so hard and fast as everyone else.<br><br>*(GP Group 3)*
Placebogenic practice it is hard to decide	Participants cannot decide whether a placebogenic practice is acceptable or unacceptable. They can list advantages as well as disadvantages close together and find it difficult to come down on one side or the other. There can be evidence of dilemmatic thinking and/or indecision.	*But I think you have got to be so careful who you target that at, so careful, because the breach of trust and that feeling of breach of trust, can have worse effects I think than the positive effect, if you see what I mean, so it is a balancing act.*<br><br>(Patient group 1)

For peer review only

Reporting checklist for qualitative study.

Based on the SRQR guidelines.

Instructions to authors

Complete this checklist by entering the page numbers from your manuscript where readers will find each of the items listed below.

Your article may not currently address all the items on the checklist. Please modify your text to include the missing information. If you are certain that an item does not apply, please write "n/a" and provide a short explanation.

Upload your completed checklist as an extra file when you submit to a journal.

In your methods section, say that you used the SRQRreporting guidelines, and cite them as:

O'Brien BC, Harris IB, Beckman TJ, Reed DA, Cook DA. Standards for reporting qualitative research: a synthesis of recommendations. Acad Med. 2014;89(9):1245-1251.

For peer review only

		Reporting Item	Page Number
**Title**			
	#1	Concise description of the nature and topic of the study identifying the study as qualitative or indicating the approach (e.g. ethnography, grounded theory) or data collection methods (e.g. interview, focus group) is recommended	1

Abstract

	#2	Summary of the key elements of the study using the abstract format of the intended publication; typically includes background, purpose, methods, results and conclusions	2

Introduction

Problem formulation	#3	Description and signifcance of the problem / phenomenon studied: review of relevant theory and empirical work; problem statement	3
Purpose or research question	#4	Purpose of the study and specific objectives or questions	3

Methods

Qualitative approach and research paradigm	#5	Qualitative approach (e.g. ethnography, grounded theory, case study, phenomenolgy, narrative research) and guiding theory if appropriate; identifying the research paradigm (e.g. postpositivist, constructivist / interpretivist) is also recommended; rationale. The rationale should briefly discuss the justification for choosing that theory, approach, method or technique rather than other options available; the assumptions and limitations implicit in those choices and how those choices influence study conclusions and transferability.	4-6

For peer review only

		As appropriate the rationale for several items might be discussed together.	
Researcher characteristics and reflexivity	#6	Researchers' characteristics that may influence the research, including personal attributes, qualifications / experience, relationship with participants, assumptions and / or presuppositions; potential or actual interaction between researchers' characteristics and the research questions, approach, methods, results and / or transferability	3
Context	#7	Setting / site and salient contextual factors; rationale	4
Sampling strategy	#8	How and why research participants, documents, or events were selected; criteria for deciding when no further sampling was necessary (e.g. sampling saturation); rationale	4
Ethical issues pertaining to human subjects	#9	Documentation of approval by an appropriate ethics review board and participant consent, or explanation for lack thereof; other confidentiality and data security issues	3-4
Data collection methods	#10	Types of data collected; details of data collection procedures including (as appropriate) start and stop dates of data collection and analysis, iterative process, triangulation of sources / methods, and modification of procedures in response to evolving study findings; rationale	4-6

For peer review only

Data collection instruments and technologies	#11	Description of instruments (e.g. interview guides, questionnaires) and devices (e.g. audio recorders) used for data collection; if / how the instruments(s) changed over the course of the study	4-6
Units of study	#12	Number and relevant characteristics of participants, documents, or events included in the study; level of participation (could be reported in results)	6
Data processing	#13	Methods for processing data prior to and during analysis, including transcription, data entry, data management and security, verification of data integrity, data coding, and anonymisation / deidentification of excerpts	6
Data analysis	#14	Process by which inferences, themes, etc. were identified and developed, including the researchers involved in data analysis; usually references a specific paradigm or approach; rationale	6
Techniques to enhance trustworthiness	#15	Techniques to enhance trustworthiness and credibility of data analysis (e.g. member checking, audit trail, triangulation); rationale	6
**Results/findings**			
Syntheses and interpretation	#16	Main findings (e.g. interpretations, inferences, and themes); might include development of a theory or model, or integration with prior research or theory	7

For peer review only

Links to empirical data	#17	Evidence (e.g. quotes, field notes, text excerpts, photographs) to substantiate analytic findings	7
**Discussion**			
Intergration with prior work, implications, transferability and contribution(s) to the field	#18	Short summary of main findings; explanation of how findings and conclusions connect to, support, elaborate on, or challenge conclusions of earlier scholarship; discussion of scope of application / generalizability; identification of unique contributions(s) to scholarship in a discipline or field	11-12
Limitations	#19	Trustworthiness and limitations of findings	12
**Other**			
Conflicts of interest	#20	Potential sources of influence of perceived influence on study conduct and conclusions; how these were managed	1
Funding	#21	Sources of funding and other support; role of funders in data collection, interpretation and reporting	1

For peer review only

The SRQR checklist is distributed with permission of Wolters Kluwer © 2014 by the Association of American Medical Colleges. This checklist was completed on 04. June 2019 using https://www.goodreports.org/, a tool made by the EQUATOR Network in collaboration with Penelope.ai

BMJ Open

Placebos in Primary Care? A Nominal Group Study Explicating UK GP and Patient Views of Six Theoretically Plausible Models of Placebo Practice

Journal:	*BMJ Open*
Manuscript ID	bmjopen-2019-032524.R1
Article Type:	Original research
Date Submitted by the Author:	15-Nov-2019
Complete List of Authors:	Ratnapalan, Mohana; University of Southampton, Primary Care and Population Sciences<br>Coghlan, Beverly; ACT Works Ltd<br>Tan, Mengxin; London School of Hygiene and Tropical Medicine, Centre of Global Mental Health; Institute of Psychiatry Psychology and Neuroscience<br>Everitt, Hazel; University of Southampton, Primary Care and Population Sciences, Faculty of Medicine<br>Geraghty, Adam; University of Southampton, Primary Care and Population Sciences<br>Little, Paul; University of Southampton, Primary Care and Population Science;<br>Lewith, George; University of Southampton, Primary Care and Population Sciences<br>Bishop, Felicity; University of Southampton, Centre for Clinical and Community Applications of Health Psychology; University of Southampton, Primary Care and Population Sciences
**Primary Subject Heading**:	General practice / Family practice
Secondary Subject Heading:	Qualitative research
Keywords:	PRIMARY CARE, QUALITATIVE RESEARCH, placebos, placebo effects, general practice

SCHOLARONE™
Manuscripts

I, the Submitting Author has the right to grant and does grant on behalf of all authors of the Work (as defined in the below author licence), an exclusive licence and/or a non-exclusive licence for contributions from authors who are: i) UK Crown employees; ii) where BMJ has agreed a CC-BY licence shall apply, and/or iii) in accordance with the terms applicable for US Federal Government officers or employees acting as part of their official duties; on a worldwide, perpetual, irrevocable, royalty-free basis to BMJ Publishing Group Ltd ("BMJ") its licensees and where the relevant Journal is co-owned by BMJ to the co-owners of the Journal, to publish the Work in this journal and any other BMJ products and to exploit all rights, as set out in our licence.

The Submitting Author accepts and understands that any supply made under these terms is made by BMJ to the Submitting Author unless you are acting as an employee on behalf of your employer or a postgraduate student of an affiliated institution which is paying any applicable article publishing charge ("APC") for Open Access articles. Where the Submitting Author wishes to make the Work available on an Open Access basis (and intends to pay the relevant APC), the terms of reuse of such Open Access shall be governed by a Creative Commons licence – details of these licences and which Creative Commons licence will apply to this Work are set out in our licence referred to above.

Other than as permitted in any relevant BMJ Author's Self Archiving Policies, I confirm this Work has not been accepted for publication elsewhere, is not being considered for publication elsewhere and does not duplicate material already published. I confirm all authors consent to publication of this Work and authorise the granting of this licence.

For peer review only

Full Title: Placebos in Primary Care? A Nominal Group Study Explicating UK GP and Patient Views of Six Theoretically Plausible Models of Placebo Practice

Authors: Mohana Ratnapalan[1], Beverly Coghlan[2], Mengxin Tan[3,4], Hazel Everitt[1], Adam W A Geraghty[1], Paul Little[1], George Lewith[1], Felicity L Bishop[1,5]

Affiliation:
1. Primary Care and Population Sciences, University of Southampton, Aldermoor Health Centre, Aldermoor Close, Southampton, SO16 5ST
2. ACTWorks Ltd 31b North Street, Emsworth, PO18 8UF
3. London School Hygiene and Tropical Medicine, Centre of Global Mental Health, Keppel Street, Bloomsbury, London, WC1E 7HT
4. Institute of Psychology, Psychiatry and Neuroscience, King's College London 6 De Crespigny Park, Camberwell, London SE5 8AB, UK
5. Centre for Clinical and Community Applications of Health Psychology, Faculty of Environmental and Life Sciences, Building 44 Highfield Campus, University of Southampton, Southampton SO17 1BJ

Corresponding author: Dr Felicity L Bishop
Centre for Clinical and Community Applications of Health Psychology, Faculty of Environmental and Life Sciences, Building 44 Highfield Campus, University of Southampton, Southampton SO17 1BJ
Phone: +44 (0)23 8059 9020 Email: F.L.Bishop@soton.ac.uk

Declarations:

Guarantor: FB

Competing interests: none

Funding: The project "Creating a Taxonomy to Harness the Placebo effect in UK primary care" was funded by the National Institute of Health Research (NIHR) School for Primary Care Research (SPCR) (project number 161). Additional funding for BC was provided by Solent NHS Trust. MR also received funding for part of her research time from the SPCR.

Disclaimer: This paper presents independent research funded by the National Institute of Health Research (NIHR). The views expressed are those of the author(s) and not necessarily those of the NHS, the NIHR, HEE or the Department of Health. The funders had no role in design and conduct of the study; collection, management, analysis, and interpretation of the data; preparation, review, or approval of the manuscript; and decision to submit the manuscript for publication.

Ethical approval: Ethical approval for this study was obtained from the Faculty of Medicine Ethics Committee, University of Southampton (reference number: 4741). Participants gave their informed consent before taking part.

Data sharing: Qualitative coding data available in appendix 1. Further quotations are available, subject to ethical approval, by contacting the authors.

Patient consent for publication: Not required

Author Contributions: FB, GL, PL, BC, HE and AG conceived and designed the study. BC conducted the nominal group meetings. FB, BC, HE, MT and MR were involved in the data analysis and interpretation. MR led the integrated analysis and drafting of the manuscript. All authors contributed to the drafting of the manuscript and take responsibility of the integrity of the data and the analysis.

Acknowledgements: We would like to thank all our participants who gave their time to partake in our nominal groups. We would also like to thank the Primary Care Research Network.

For peer review only - http://bmjopen.bmj.com/site/about/guidelines.xhtml

Abstract

Objectives: To better understand which theoretically plausible placebogenic techniques might be acceptable in UK primary care.

Design: A qualitative study using nominal group technique and thematic analysis. Participants took part in audio-recorded face-to-face nominal groups in which the researcher presented 6 scenarios describing the application in primary care of theoretically plausible placebogenic techniques: (1) Withholding side-effects; (2) Monitoring (3) GP endorsement (4) Idealised consultation (5) Deceptive placebo pills (6) Open-label placebo pills. Participants voted on whether they thought each scenario was acceptable in practice and discussed their reasoning. Votes were tallied and discussions transcribed verbatim.

Setting: Primary care in England.

Participants: 21 GPs in 4 nominal groups and 20 "expert patients" in 5 nominal groups.

Results: Participants found it hard to decide which practices were acceptable and spoke about needing to weigh potential symptomatic benefits against the potential harms of lost trust eroding the therapeutic relationship. Primary care patients and doctors felt it was acceptable to harness placebo effects in practice by patient self-monitoring (scenario 2), by the GP expressing a strongly positive belief in a therapy (3), and by conducting patient-centered, empathic consultations (4). Deceptive placebogenic practices (scenarios 1 and 5) were unacceptable to most groups. Patient and GP groups expressed a diverse range of opinions about open label placebo pills.

Conclusions: Attempts to harness placebo effects in UK primary care are more likely to be accepted and implemented if they focus on enhancing positive patient-centered communication and empathic relationships. Using placebos deceptively is likely to be unacceptable to both GPs and patients. Open-label placebos also do not have clear support; they might be acceptable to some doctors and patients in very limited circumstances - but further evidence, clear information and guidance would be needed.

Keywords: placebos, placebo effects, primary care, general practice, qualitative research

Article Summary: Strengths and limitations of this study

- Nominal group technique and thematic analysis was used to identify key opinions from both GPs and patients on theoretically plausible placebogenic techniques Participants were recruited through research networks and patient charities and sampled to achieve a broad range of views
- Scenarios discussed were carefully constructed to reflect potential placebogenic practice based on clinical and experimental research evidence
- Some nominal groups were small due to the availability of participants.
- It was not always possible to achieve a clear majority opinion on the scenarios

For peer review only - http://bmjopen.bmj.com/site/about/guidelines.xhtml

Manuscript (Word count 3806/4000)

Background

Placebos have an uncertain role in everyday medical practice. They have a long history [1-3] and evidence suggests therapeutic benefit [4-10]. However, there is no broad consensus on how to define placebos nor the ethics of use in clinical practice [11, 12]. Definitions vary between placebos as a substance, a process (e.g. practitioner empathy) or both [11]. This paper defines placebos as substances or processes other than the active ingredients of treatment, which can have substantial effect on symptoms. We define placebo effects as beneficial symptomatic changes triggered by the meaning a person experiences in a healthcare setting [13, 14].

In the UK, there are over 300 million primary care consultations annually [15] with rising demand in the last decade [16]. Within this context, it becomes important to optimise doctor-patient encounters for maximum health benefit. Placebogenic practices, i.e. techniques that can trigger placebo responses in clinical settings, could support cost-effective healthcare, which minimises patient harm from drug side effects and/or enhances the effects of prescribed evidence-based therapies. A recent meta-analysis describes frequent use of placebos in primary care with particularly high use of non-specific therapies (E.g. physician as placebo to exert positive psychological effect) [17]. A meta-ethnographic review of patient and doctor views on placebo practice found acceptable use to patients include therapeutic benefit and giving hope; with health care professionals also citing therapeutic benefit and placebos as clinical management tools [11]. However, few studies directly compared doctors' and patients' views. A meta-analysis on open-label placebos, where patients are honestly informed they are being given placebos found positive clinical effects [18]. However, few qualitative studies have explored patients' or doctors' perspectives on open-label placebos [19]. We used nominal group technique [20-22], a qualitative consensus building technique, to explore and compare how patients and doctors conceive a range of placebogenic practices and why certain practices are more acceptable.

Methods

We gained ethical approval from the Faculty of Medicine Ethics Committee, University of Southampton (reference number: 4741).

Our research team consists of health psychologists (FB, AG), GPs (PL, GL, HE), a psychotherapist (BC), a psychology student (MT) and a GP Academic Clinical Fellow (MR). This range of backgrounds enriched our qualitative analysis, enabling us to bring diverse perspectives to the data and ensuring we explored multiple potential themes, remained open to new ways of conceptualising the data, worked to evidence our interpretations in the raw data, and avoided an idiosyncratic interpretation. Ultimately our approach to data collection and analysis was driven by our pragmatic aim to examine which placebogenic practices might be more or less acceptable to patients and GPs and why.

For peer review only - http://bmjopen.bmj.com/site/about/guidelines.xhtml

Patient and Public Involvement

No patient involved. We did not specifically involve patients or the public in the design, conduct or reporting of this study. However, this study aimed to capture patient views.

Participant Recruitment

We recruited English-speaking GPs and adult "expert patients", i.e. those with recent experience of using health services and involvement in patient organizations or medical research. We advertised to GPs through the south-west primary care research network and to patients through UK-wide patient associations and health charities (e.g. Pain UK, CFS/ME groups). Individuals expressing interest were mailed a participant information sheet and completed a consent form before participation. We deliberately sought to include GPs and patients of a range of ages and genders and patients with a range of health conditions.

Participants who agreed to participate did so on pre-specified days. The number of people willing to participate determined group size and composition.

Nominal Groups

We structured nominal group meetings as per methodological guidance [20, 22](Table 1). BC, an accredited psychotherapist experienced in facilitation, led the group meetings held in suitable venues (e.g. meeting rooms in GP practices) between April and August 2013.

Table 1: Nominal Group Meeting Structure

Phase	Activity
**Informed Consent**	Facilitator (BC) talks through participant information sheets and consent forms and answers any questions. Participants sign consent forms.
**1: Introduction**	Facilitator introduces the topic, explains our interest in it and asks participants to introduce themselves
**2: Silent reflection**	Participants read the scenario and write comments
**3: Round robin**	Facilitator elicits one comment from each participant and writes this on a flipchart. Discussion not allowed. Continues until comments exhausted.
**4: Discussion**	Facilitator guides discussion of comments on each scenario in turn, using open-ended questions and ensuring all participants had the opportunity to contribute their perspective.
**5: Voting**	Participants vote whether the scenario is acceptable or not. (Undecided was also permitted)
**6: Repeat**	Processes 2 to 5 are repeated in turn for each scenario
**7: Break**	Facilitator counts votes
**8: Discussion**	Results of votes presented and discussed. Each scenario without clear majority is discussed in turn.
**9: Voting**	Second round of voting if no clear majority in first round of voting.
**10: Conclusion**	Results of vote. Facilitator explains future plans and thanks participants.

Nominal groups were presented 6 scenarios for voting and discussion. These scenarios were written by the research team using a taxonomy of five domains of placebogenic techniques [23-27] derived

For peer review only - http://bmjopen.bmj.com/site/about/guidelines.xhtml

from experimental and clinical studies [28] to create 6 theoretically plausible placebogenic scenarios for primary care (Table 2). Techniques from the five domains [23] were used to create the scenarios: 1) the patient's beliefs and characteristics informed 'Withholding Side-Effects'; 2) The healthcare setting informed 'Monitoring'; 3) The practitioner's beliefs and characteristics informed 'GP endorsement'; 4)the patient-practitioner interaction informed 'Idealised consultation'. 5) Treatment characteristics informed 'Deceptive'/'Open-label Placebo Pills'. GP groups read scenarios written from the GP perspective.

Table 2: Scenarios for Patient Groups

Scenario	Aspect that might enhance placebo responding
**Scenario 1: "Withholding Side-Effects"**<br>You visit your GP because you have noticed new or worsened symptoms. Your GP examines you, asks you about your symptoms, gives you a diagnosis and decides to prescribe medication. Your GP knows that if she provides you with positive information about the medication you are more likely to notice a benefit. So to make you feel hopeful about your treatment she tells you, truthfully, that research has shown that the majority of patients taking this medication notice a big improvement in their symptoms, and that you too, should notice a big improvement. The medication might have side-effects, but your GP does not tell you about these. This is because she knows that if she *does* tell you about the possible side-effects then you will be more likely to suffer from them.	Giving a positive message may enhance patients' response expectancy; withholding information about medication side effects may reduce the chances of the patient developing them via nocebo mechanisms.
**Scenario 2: "Monitoring''**<br>You visit your GP because you have noticed new or worsened symptoms. Your GP advises you to continue with your usual treatment but requests that you attend the surgery more frequently for on-going review and monitoring of your condition. She also asks you to monitor your symptoms on a daily basis and report back to her at your next visit. She provides you with a special symptom-monitoring diary to help you to do this.	The use of regular monitoring and review may increase awareness of symptom changes and potentially motivate behavioural changes. .
**Scenario 3: "GP endorsement''**<br>You visit your GP because you have noticed new or worsened symptoms. Your GP examines you, asks you about your symptoms, gives you a diagnosis and offers to prescribe a particular medication. You have heard of this medication and are not sure how effective it will be and ask if there are any other treatments you could try instead. Your GP says that there are but that he strongly believes (based on his experience with other patients and from published research) that the medication he wants to prescribe provides absolutely the best chance of reducing your symptoms in the shortest time.	Conveying the clinicians' strong personal beliefs about a particular medication may enhance patients' response expectancy.
**Scenario 4: "Idealised consultation''**<br>You visit your GP because you have noticed new or worsened symptoms. Your appointment is with the same GP you always see. He greets you warmly and seems pleased to see you. He turns away from his computer screen and gives you his full attention. He is very interested and concerned about what you tell him. He asks you many detailed questions about how the symptoms started and how they are now affecting your daily life. He thoroughly examines you. He genuinely seems to be interested in you as a person and not as just a collection of symptoms. He allows you time to ask questions and even though he does not know all of the answers he provides as much information as he can and says he will try to find out more and will get back to you later in the day by telephone. He tells you that he would prefer it if you continue to make your appointments with him in future.	Enhanced attention, more time, warm and empathic and collaborative style may enhance perception of empathy, validation, and response expectancy.

For peer review only - http://bmjopen.bmj.com/site/about/guidelines.xhtml

**Scenario 5: "Deceptive placebo pills''**<br>You attend your GP surgery because you have noticed new or worsened symptoms. Your GP examines you, asks you about your symptoms, gives you a diagnosis and recommends a prescription for medication. She tells you that research has shown that by taking this medication three times a day for at least a week, your symptoms will get better. What she **does not** tell you is that the medication she will be prescribing is actually a "placebo" pill that contains **no real medicine.**	Prescribing a placebo medication deceptively may enhance response expectancy and engender a conditioned response to pill taking.
**Scenario 6: " Open-label placebo pills''**<br>You attend your GP surgery because you have noticed new or worsened symptoms. Your GP examines you, asks you about your symptoms, gives you a diagnosis and recommends a prescription for medication. She tells you that research has shown that by taking this medication three times a day for at least a week, your symptoms will get better. What she **does** tell you is that the medication she will be prescribing is actually a "placebo" pill that contains **no real medicine.**	Prescribing a placebo medication openly may enhance response expectancy and engender a conditioned response to pill taking.

Data Analysis

Each meeting was audio-recorded, transcribed verbatim and anonymized.

Our analytical approach rested on principles described by McMillian et al and encompassed attending to both participants' votes and qualitative discussions [21]. Votes were counted and each group was classified according to whether the majority of participants deemed each scenario acceptable, unacceptable, or 'no clear majority'. To analyse the discussions we used thematic analysis [29] with constant comparison between groups and scenarios. After repeated reading of transcripts, initial low-level inductive codes were developed independently for the GP and patient transcripts by MR and MT respectively, using Nvivo 12 to facilitate coding and maintain an audit trail. Low level codes were reviewed by FB, MR, and MT who iteratively developed higher level codes by merging similar low-level codes and combining them into a hierarchical structure. MR led the search for themes by comparing and contrasting codes across scenarios and across GP and patient groups, reviewing potential themes for fit with the raw data. MR, HE, and FB discussed which themes best captured GPs' and patients' reasoning around placebogenic practice and agreed on the final 16 themes (see Appendix 1). MR then integrated the qualitative themes with the vote frequency data using an iterative process comparing votes and themes (a) within groups across individual scenarios and (b) within scenarios across groups. This analysis was developed and agreed by all authors and is presented below. We used the SRQR checklist when writing our report[30].

Results

Participant characteristics

21 GPs and 20 patients (Table 3) participated in 9 nominal groups (4 GP and 5 patient groups); with 2 to 8 participants and lasting 75-100 minutes per group. Most GPs (n=17, 81%) were working full-time. 2 patients completed sixth form or college, 4 university undergraduate, and 9 post-graduate (5 did not disclose). 15 patients disclosed their general health status as follows: very good, n=7 (35%),

For peer review only - http://bmjopen.bmj.com/site/about/guidelines.xhtml

good 1 (5%), fair 6 (30%), bad 1 (5%). Patients' self-reported health conditions included: chronic pain, irritable bowel syndrome, cancers and diabetes.

Table 3: Demographics

	GP	Patient
**Total n**	21	20
**Number of males (%)**	12 (57%)	7 (35%)
**Number of females (%)**	9 (43%)	13 (65%)
**Mean age (SD) ****	42 (9.2)	56.3 (12.7)
**Mean years GP (SD)*****	15 (10.1)	-
**Range of group size (mean)**	3-8 (5)	2 – 7 (4)

Notes: ** 5 not disclosed; * 3 not disclosed. Undisclosed demographic data comes from different nominal groups and is not isolated missing data for any single group.**

Qualitative Analysis

Overview

Participants found it hard to decide whether each placebogenic practice was acceptable. Patients and GPs spoke about the tension between balancing positive effects of placebogenic practice against harmful erosion of the therapeutic relationship from loss of trust.

''But I think you have got to be so careful … because the breach of trust and that feeling of breach of trust, can have worse effects I think than the positive effect… so it is a balancing act.''

(Patient Group 1)

''… the nice thing about GPs is having the ongoing patient relationship. So we' re also trying to build a relationship and that's, obviously, part of a placebo effect. But if you tell patients it's going to work brilliantly and it doesn't then that slightly damages their trust, versus if you tell them that they might get a side-effect but it will settle down… But again, it's either damaging or enhancing in the GP relationship, as well.''

(GP Group 3)

Despite these tensions, there were some consistent patterns in the voting (Table 4). 'Monitoring' and 'GP endorsement' were acceptable to all GP groups while the 'Idealised consultation' was acceptable to all GP and patient groups. The arguments that participants offered in the discussions to justify their votes are explored below.

Table 4: Tabulated group level voting on acceptability of 6 scenarios of placebogenic practice

Scenario	Acceptable	No clear majority	Unacceptable
1. "Withholding side effects"	Δ Δ	Δ Δ ◯	Δ ◯◯◯
2. "Monitoring"	Δ Δ Δ Δ ◯ ◯◯◯	Δ	
3. "GP endorsement"	Δ Δ Δ Δ	Δ	

For peer review only - http://bmjopen.bmj.com/site/about/guidelines.xhtml

	◯ ◯ ◯ ◯		
4. "Idealised consultation"	Δ Δ Δ Δ Δ ◯ ◯ ◯ ◯		
5. "Deceptive placebo pills"		Δ	Δ Δ Δ Δ ◯ ◯ ◯ ◯
6. "Open-label placebo pills"	◯ ◯	Δ Δ Δ ◯	Δ Δ ◯

◯ = GP groups (n=4) Δ = Patient groups (n=5)

For peer review only

GPs and patients felt that "Monitoring" empowered patients by providing patient centred care. GPs argued that involving the patient and using time as diagnostic tool could help them consult more effectively, but expressed concern that over-emphasising symptoms could lead to psychological harms (e.g. generating anxiety). The acceptability of this scenario was felt to depend on the medical condition, the patient's characteristics (e.g. age), the work required of the patient for self-monitoring, the disease process and the symptom severity.

"..it would also provide me with more control …over my condition… by being aware of change."

(Patient Group 5)

"Potentially, I'll say, I would do this – if, in the 10 minutes I've got available, I really haven't got a true reflection of you know, what the symptom pattern is and what the effect on the patient really is, then it's just a way of extending the consultation over a period of time and to actually gather that information."

(GP Group 2)

GP groups discussed how the GPs' experience and the evidence-base would influence the acceptability of "GP endorsement". GPs felt there needed to be a published evidence-based benefit or personal experience of likely therapeutic benefit to endorse a therapy. Patients felt that "GP endorsement" might be more acceptable in the context of more egalitarian doctor-patient relationships.

"Again I think it depends on the relationship I as the patient had with that GP, whether it was a relationship I felt was equal, or not. If it was then I would be more inclined to go along with that advice. If I felt it was more of a paternalistic relationship than I would be questioning why, why does he think this is the best one. I'd want more information about that drug, and also to discuss whatever it is that I've heard about this medication and why I've heard it's not necessarily the best thing. And also to be sure that it's not being prescribed because it's the latest drug that pharmaceuticals are pushing and this is a really good one and it will do all singing, all dancing."

(Patient Group 2)

The continuity of care within the "Idealised consultation" was particularly well-received. GPs felt continuity of care enhanced their job satisfaction and improved their understanding the

psychosocial context of their patients by permitting long-term relationships to develop with patients and families. GPs felt continuity provided a directed trajectory of care that disjointed multi-practitioner led care might not provide. Patients agreed and valued the idea of seeing a practitioner who knew their story.

Despite universal acceptance of the "Idealised consultation", GPs and patients also expressed concerns about this. GPs were concerned that knowing their patients too well could lead to harm from cognitive bias and encourage patients to become overly reliant on one doctor and subsequently come to harm from delaying presenting if that doctor was unavailable. GPs and patients both expressed concerns that this scenario was unrealistic given workloads and/or would increase GP workload, which may in turn negatively affect care, and that this type of practice could blur doctor-patient boundaries.

''And sometimes you know your patient so well that you just don't see that they're losing weight or they're becoming hypothyroid or something.''

(GP Group 4)

'' [F1]: Well, I say a GP from heaven! …I would have full trust and confidence in the GP, if ever they had that sort of response to you and a welcoming aspect to it, and naturally being eye-contact, focussing with you, and receptive both ways. And interested in you and there is communication, as a key factor. And to be able to leave that surgery knowing that you have got some form of support out there, in such an isolating situation whenever you are in chronic pain.

[F3]: I mean I think even the admission that he doesn't know all the answers is reassuring, because GPs are what GPs are, they are not specialists, they have to know something about a lot of things, but not necessarily deep down into one specialisation, but they know where to go….''

(Patient Group 1)

Deception in Placebogenic Practice

"Withholding side-effects" and "Deceptive placebo pills" both involve deception. Most groups found "Withholding side effects" unacceptable or impossible to reach a majority judgement, while all but one group found "Deceptive placebo pills" unacceptable.

GPs and patients were worried about the risks of physical and psychological harm and damage to the GP-patient relationship from withholding information about side- effects. For example, one GP group was concerned about patient harm from an accident if they were unaware of potential impaired function. One patient group discussed how an unexpected side-effect might cause anxiety that this was a new health problem. Patients felt that withholding information disempowers them and being inconsistent with patient-centred care where ultimate autonomy rests with the patient to make informed decisions. GP groups also discussed medico-legal and policy issues. They worried about medico-legal implications of non-disclosure and discussed how government targets may alter

For peer review only - http://bmjopen.bmj.com/site/about/guidelines.xhtml

their discussions about medication. However, patients were more accepting of ‘Withholding side effects’ than the GP groups. The patient groups who found this to be acceptable practice spoke about GPs knowing their patients and using their judgement on when it might be permissible to not mention side effects based on having an effective partnership built on trust with their GP.

‘’Yes, I think my views change with time, too, and the outside world we’re working in. I’m far more likely to give somebody an ACE inhibitor now than I was five years ago, simply because its part of the QOF targets. And that has a big effect on the way I will sell an ACE, give an ACE and encourage the patient to use it. I probably use quite a lot of the ‘Doctor knows best’ concept in the consultation to push that particular drug. Because there is a monetary target involved with it.’’

(GP Group 3)

‘’To me, it depends upon the frequency and the severity of the side effects. Because if they’re rare and minor I would be completely comfortable with it, if they’re serious or very frequent I’d be uncomfortable with it because you risk loss of trust…. And again, there’s the risks, especially if … it impaired their function and they had an accident or, you know, there’s risks to not telling people about potential side effects.’’

(GP Group 1)

‘’Yes. If you’re expecting a patient to take a drug then they have to understand potentially what the problems or issues could be. I mean, you know, even if they are fairly minor I think most people understand that it’s only a 1% chance of things happening, but at least it’s their decision to take that drug or to take that treatment, and they can’t take that decision if they’re being pushed, if you like, being pushed or being persuaded to do it if they don’t get the full information and it’s not the doctor’s decision, it’s the patient’s decision.’’

(Patient Group 4)

In contrast, “Deceptive placebo pills” involved more active deception and was felt to be dishonest. GPs were concerned that this was ethically unacceptable practice and this drove their decision-making irrespective of potential benefit from placebo pills. GPs felt that it was imperative that patients were able to make fully informed decisions about therapy in this scenario. There was fear of repercussions from the General Medical Council, as the use of placebo pills is not, currently, incorporated into professional codes of practice nor accepted within the wider cultural context of medical practice. One GP group felt there might be a role for deceptive placebo pills in (unspecified) mental illness. However, the same group expressed a tension between personal ethics and accepted codes or standards of practice.

Similarly, patients expressed discomfort with receiving unknown substances and judged the deceit involved as ethically unacceptable. Patients spoke of risk of psychological harm from feeling that their symptoms were not ‘real’ and were “all in the mind”, with some seeing placebo pills as not real therapy for real symptoms. They also worried that placebo pills would be a way for GPs to avoid properly investigating their problems. Patients spoke of subverting the placebogenic effect by seeking information about the pills outside the consultation (e.g. online). Both patients and GPs expressed concerns about deceptive prescribing eroding the doctor-patient relationship.

‘’[M1]: Do you believe in a placebo?

For peer review only - http://bmjopen.bmj.com/site/about/guidelines.xhtml

[F1]: …. I do believe in it but that's not actually what's being asked…I believe that the patient should know what it is they're signing up to. So I'm really happy in a proper clinical trial where you're told you could go into the placebo arm or you could go into the arm where you will get the drug. That's absolutely fully acceptable and you then don't know whether you've got the placebo. That's great, but this is wrong, this is underhand.

[F2]: I think it's the GP judging you, thinking he or she knows you really well. I mean how well do they know you from a 5-10 minute consultation. You know, have they asked you about other things going on in your life? Other issues and things, or are they just focussing on that one aspect.

[F1]: Yeah, give them a dummy pill and then they'll go away and be quiet. As opposed to actually, you know, getting down to what the issue actually is.''

(Patient Group 4)

''And how do you feel about that? How do you defend against that? And what is the patient going to think of you next time they see you, if they realise it was a fake pill, so to speak? And how do they have confidence in you from thereon in?''

(GP Group 2)

''I could live with it ethically, I think the problem is the GMC code of practice, isn't it?''

(GP Group 1)

Open-label placebo pills

In contrast to "Deceptive placebo pills", "Open-label placebo pills" removed the element of deception with placebo pills. Despite many of the objections to "Deceptive placebo pills" focusing on the deception per se, removing the deceptive element did not lead to a complete shift towards groups seeing placebos as acceptable. Although GPs groups were more accepting of this scenario, patients felt that the acceptability of open-label placebo depended on the medical condition and their trust in the GP. Patients were not happy to pay a prescription charge for what they saw as an inert pill and if they saw placebo as inappropriate or ineffective they argued that this would weaken the therapeutic effect.

''That it was a placebo and that it was found to work in other people, I'd think great, I'll give it a go, yeah. I'd be quite happy about that, it's the not being told that I have the problem with.''
(Patient Group 2)

Some GPs felt that prescribing alone was not enough and additional positive talk and a cultural shift would be required. Others worried that patients would stop seeing them if they prescribed placebo pills.

*''**M1** … I've never personally done that but I know when I was doing paediatrics there was a child with quite profound functional symptoms …and they knew they were having oral saline and they got better, they improved. So I would be more comfortable with that but I've never had a clinical context where I've had the courage to do it but if it …*

***F2** Me neither.*

***M1** But if it was more of a sort of cultural thing I would be very glad we're doing that.''*

For peer review only - http://bmjopen.bmj.com/site/about/guidelines.xhtml

(GP Group 4)

"I just think it's mad. If I did that with my patients they'd never come and see me again and say, "I'll get a doctor that gives me actual medicine."

(GP Group 1)

Discussion

Our study captures both GP and patient views and offers new insights on the real-world application of placebos. We found that placebogenic practice with deception is very clearly not acceptable and for open-label placebo pills, there was no clear judgement of acceptability from any of the patient groups. This extends on previous studies, which suggest that GPs found deceptive placebogenic practice unacceptable [31, 32] and some patients feel it is important for any placebogenic practice to respect patient autonomy [33]. By focusing on theoretically plausible placebogenic scenarios, we provide new insights to placebogenic practices with potential for implementation to enhance patient outcomes in clinical practice and clarified the psychological and sociocultural barriers that would need to be overcome.

We found the acceptability of placebogenic practice is difficult to determine and even 'acceptable' scenarios elicited talk of caveats, as did a recent meta-ethnography [11]. Caveats to acceptability identified in our study include, but are not limited to, considerations of: medical condition; individual patient; individual doctor; regulatory norms; government prescribing targets; GMC guidance and what is viewed as acceptable practice to other medical colleagues (i.e., social norms). This suggests that any generic guidelines proclaiming a type of practice as either 'acceptable' or 'unacceptable' may not capture the views of GPs and patients as key stakeholders and may be problematic. It may be more useful to develop guidance that highlights important considerations and contexts for placebogenic practice.

Our study is limited by non-blinded voting. The group method means discussions must be interpreted in their social and cultural context, and not as individuals' personal beliefs. It is informative, although not surprising, that the GP groups discussed clinical practice norms while the patient groups (comprising expert patients who were typically accustomed to acting as patient advocates) drew heavily on the notion of patient autonomy. Indeed, the composition of our patient groups must be considered when interpreting our findings. By deliberately seeking "expert patient" participants we have gained insight into how particularly engaged and politically aware patients with high health literacy discuss placebos in general practice. Had we sought a more diverse sample of patients including for example those with lower health literacy and less engagement with health services then different issues may have emerged as important in the patient group discussions. Despite attempts to purposively sample a diverse group, some nominal groups were small with 2 or 3 participants. Our findings may also be limited by the sequence in which cases were presented to groups. Participant views on open-label placebo may have been influenced by preceding discussions of placebo pills prescribed deceptively. However, it was felt important to present scenarios in a way that would encourage discussion and offer participants a "way in" to this complex topic, hence we chose to present the more familiar examples of deceptive placebo prescribing before moving on to explore open label placebo. Findings indicate patterns of views held by our participants, all of whom

For peer review only - http://bmjopen.bmj.com/site/about/guidelines.xhtml

volunteered their time and so might be more interested in and/or hold stronger views about placebo effects than non-participants.

Conclusions and Future Work

Our study helps inform future work on placebogenic practice and provides clinicians with improved understanding of what peers and patients would find acceptable, whilst acknowledging this is a complex area with diverse opinions. Our study suggests that open-label placebo pills are not fully acceptable and future translational work could consider prescription costs among other issues. Additional research evaluating acceptable placebogenic techniques in clinical settings is needed to help inform clinicians about the effectiveness of these techniques in clinical practice.

For peer review only

For peer review only - http://bmjopen.bmj.com/site/about/guidelines.xhtml

References

1. Booth C. The rod of Aesculapios: John Haygarth (1740-1827) and Perkins' metallic tractors. *Journal of medical biography* 2005; 13: 155-161. 2005/08/02. DOI: 10.1177/096777200501300310.
2. Czerniak E and Davidson M. Placebo, a historical perspective. *European neuropsychopharmacology : the journal of the European College of Neuropsychopharmacology* 2012; 22: 770-774. 2012/05/23. DOI: 10.1016/j.euroneuro.2012.04.003.
3. Jutte R. The early history of the placebo. *Complementary therapies in medicine* 2013; 21: 94-97. 2013/03/19. DOI: 10.1016/j.ctim.2012.06.002.
4. Zhang W, Robertson J, Jones AC, et al. The placebo effect and its determinants in osteoarthritis: meta-analysis of randomised controlled trials. *Annals of the rheumatic diseases* 2008; 67: 1716-1723. 2008/06/11. DOI: 10.1136/ard.2008.092015.
5. Kirsch I, Deacon BJ, Huedo-Medina TB, et al. Initial severity and antidepressant benefits: a meta-analysis of data submitted to the Food and Drug Administration. *PLoS medicine* 2008; 5: e45. 2008/02/29. DOI: 10.1371/journal.pmed.0050045.
6. Patel SM, Stason WB, Legedza A, et al. The placebo effect in irritable bowel syndrome trials: a meta-analysis. *Neurogastroenterology and motility : the official journal of the European Gastrointestinal Motility Society* 2005; 17: 332-340. 2005/05/27. DOI: 10.1111/j.1365-2982.2005.00650.x.
7. Howick J, Friedemann C, Tsakok M, et al. Are treatments more effective than placebos? A systematic review and meta-analysis. *PloS one* 2013; 8: e62599. 2013/05/22. DOI: 10.1371/journal.pone.0062599.
8. Charlesworth JEG, Petkovic G, Kelley JM, et al. Effects of placebos without deception compared with no treatment: A systematic review and meta-analysis. *Journal of evidence-based medicine* 2017; 10: 97-107. 2017/04/30. DOI: 10.1111/jebm.12251.
9. Kaptchuk TJ, Friedlander E, Kelley JM, et al. Placebos without deception: a randomized controlled trial in irritable bowel syndrome. *PloS one* 2010; 5: e15591. 2011/01/05. DOI: 10.1371/journal.pone.0015591.
10. Hrobjartsson A and Gotzsche PC. Placebo interventions for all clinical conditions. *The Cochrane database of systematic reviews* 2010: Cd003974. 2010/01/22. DOI: 10.1002/14651858.CD003974.pub3.
11. Hardman DI, Geraghty AW, Lewith G, et al. From substance to process: A meta-ethnographic review of how healthcare professionals and patients understand placebos and their effects in primary care. *Health (London, England : 1997)* 2018: 1363459318800169. 2018/09/22. DOI: 10.1177/1363459318800169.
12. Linde K, Friedrichs C, Alscher A, et al. The use of placebo and non-specific therapies and their relation to basic professional attitudes and the use of complementary therapies among German physicians--a cross-sectional survey. *PloS one* 2014; 9: e92938. 2014/04/04. DOI: 10.1371/journal.pone.0092938.
13. Brody H. The placebo response. Recent research and implications for family medicine. *The Journal of family practice* 2000; 49: 649-654. 2000/08/03.
14. Moerman DE and Jonas WB. Deconstructing the placebo effect and finding the meaning response. *Annals of internal medicine* 2002; 136: 471-476. 2002/03/20.
15. Digital N. Appointments in General Practice, October 2018 https://digital.nhs.uk/data-and-information/publications/statistical/appointments-in-general-practice/oct-2018 (2018, accessed 08/01/2019 2019).
16. Baird B CA, Honeyman M, Maguire D, Das P. *Understanding pressures in general practice*. May 2016 2016. The King's Fund.

For peer review only

17. Linde K, Atmann O, Meissner K, et al. How often do general practitioners use placebos and non-specific interventions? Systematic review and meta-analysis of surveys. *PloS one* 2018; 13: e0202211. 2018/08/25. DOI: 10.1371/journal.pone.0202211.
18. Charlesworth JEG, Petkovic G, Kelley JM, et al. Effects of placebos without deception compared with no treatment: A systematic review and meta-analysis. *Journal of evidence-based medicine* 2017; 10: 97-107. DOI: doi:10.1111/jebm.12251.
19. Kaptchuk TJ and Miller FG. Open label placebo: can honestly prescribed placebos evoke meaningful therapeutic benefits? *BMJ (Clinical research ed)* 2018; 363: k3889. DOI: 10.1136/bmj.k3889.
20. Van de Ven AH and Delbecq AL. The effectiveness of nominal, delphi, and interacting group decision making processes. *Academy of Management Journal* 1974; 17: 605-621. DOI: 10.2307/255641.
21. McMillan SS, Kelly F, Sav A, et al. Using the Nominal Group Technique: how to analyse across multiple groups. *Health Services and Outcomes Research Methodology* 2014; 14: 92-108. journal article. DOI: 10.1007/s10742-014-0121-1.
22. McMillan SS, King M and Tully MP. How to use the nominal group and Delphi techniques. *International journal of clinical pharmacy* 2016; 38: 655-662. 2016/02/06. DOI: 10.1007/s11096-016-0257-x.
23. Di Blasi Z, Harkness E, Ernst E, et al. Influence of context effects on health outcomes: a systematic review. *Lancet (London, England)* 2001; 357: 757-762. 2001/03/20.
24. Dewey D, McDonald MK, Brown WJ, et al. The impact of ecological momentary assessment on posttraumatic stress symptom trajectory. *Psychiatry Research* 2015; 230: 300-303. DOI: https://doi.org/10.1016/j.psychres.2015.09.009.
25. Goldstein SP, Goldstein CM, Bond DS, et al. Associations between self-monitoring and weight change in behavioral weight loss interventions. *Health psychology : official journal of the Division of Health Psychology, American Psychological Association* 2019 2019/09/27. DOI: 10.1037/hea0000800.
26. Willett M, Duda J, Fenton S, et al. Effectiveness of behaviour change techniques in physiotherapy interventions to promote physical activity adherence in lower limb osteoarthritis patients: A systematic review. *PloS one* 2019; 14: e0219482. 2019/07/11. DOI: 10.1371/journal.pone.0219482.
27. Palermo S, Rainero I, Stanziano M, et al. A novel neurocognitive approach for placebo analgesia in neurocognitive disorders. *Experimental Gerontology* 2019; 118: 106-116. DOI: https://doi.org/10.1016/j.exger.2019.01.011.
28. Bishop FL, Coghlan B, Geraghty AW, et al. What techniques might be used to harness placebo effects in non-malignant pain? A literature review and survey to develop a taxonomy. *BMJ open* 2017; 7: e015516. 2017/07/02. DOI: 10.1136/bmjopen-2016-015516.
29. Braun V and Clarke V. Using thematic analysis in psychology. *Qualitative Research in Psychology* 2006; 3: 77-101. DOI: 10.1191/1478088706qp063oa.
30. O'Brien BC, Harris IB, Beckman TJ, et al. Standards for reporting qualitative research: a synthesis of recommendations. *Academic medicine : journal of the Association of American Medical Colleges* 2014; 89: 1245-1251. 2014/07/01. DOI: 10.1097/acm.0000000000000388.
31. Howick J, Bishop FL, Heneghan C, et al. Placebo use in the United kingdom: results from a national survey of primary care practitioners. *PloS one* 2013; 8: e58247. 2013/03/26. DOI: 10.1371/journal.pone.0058247.
32. Bishop FL, Howick J, Heneghan C, et al. Placebo use in the UK: a qualitative study exploring GPs' views on placebo effects in clinical practice. *Family practice* 2014; 31: 357-363. 2014/04/17. DOI: 10.1093/fampra/cmu016.
33. Bishop FL, Aizlewood L and Adams AE. When and why placebo-prescribing is acceptable and unacceptable: a focus group study of patients' views. *PloS one* 2014; 9: e101822. 2014/07/10. DOI: 10.1371/journal.pone.0101822.

For peer review only - http://bmjopen.bmj.com/site/about/guidelines.xhtml

For peer review only

For peer review only - http://bmjopen.bmj.com/site/about/guidelines.xhtml

Appendix 1: Summary of Themes

Theme	Definition	Example quote
Consultation and clinical practice behaviours	General talk which is not necessarily specific to placebogenic practice but which dictates the models of consultation and also broader consideration about working life of a GP.	*I think very few medications I prescribe actually have the best chance of reducing anyone's symptoms in a short time. The majority of medications I'm prescribing are preventative, and that's when you often have to do the sales job, because they're not going to have any change in their symptoms but you just try and give them what's needed to treat them, or I have done. So I think the preventative thing is quite interesting. But I would use the technique at times.*<br><br>(GP Group 3)
Patient perspectives on GP behaviour	General talk about the need for GPs to treat patients as individuals and how GPs should respect patients, e.g. by how they listen and talk to them. Not linked to placebogenic practice. Mainly derived from general talk in the patient groups about past experiences with GPs.	*Yes, because I think that all treatment should be a holistic approach, in that you have got to treat the whole person. It is all very well treating one condition, but sometimes when there are multiple conditions, or it is one condition that has multiple effects, you need to look at the actual person, and to me, very much, this was the approach of looking at one person, and making them feel … and they are taking part in it. And I think that is important.*<br><br>(Patient Group 1)
Placebo models	Talk that alludes to how participants think placebos might operate to generate effects on patients. How do they work, if indeed they do work? Not specifically linked to a particular placebogenic practice under discussion, rather this is more general talk	*Well this is where the placebo effect comes in – the expectation*<br><br>(Patient Group 4)

For peer review only

	about the mechanisms thought to possibly underpin placebo effects.	
Placebogenic practice – health system and medico-legal framework consideration	Aspects of the wider health system that are relevant to the acceptability of placebogenic practice e.g. GMC rules or health targets or legal framework of clinical practice	*And again, there's the risks, especially if there's, you know, it impaired their function and they had an accident or, you know, there's risks to not telling people about potential side effects.*<br><br>(GP Group 1)
Placebogenic practice – honesty, ethical practice and disclosure	Talk about the acceptability and ethical issues around honesty, deception, and complete/incomplete disclosure. About the information that "should" be provided to patients, the information that patients want, and why this is seen as important. Talk about the moral imperative for full information and informed consent, as well as occasional situations where these things are deemed non-essential. Includes talk about the circumstances in which some dishonesty or incomplete disclosure might be tolerated.	*That's unethical. So, if you've got a 1 in 10 chance that actually taking this medication is going to cause you some significant harm, we've got to be completely open about this.*<br><br>(GP Group 2)
Placebogenic practice – patient considerations	Aspects of the patient (e.g. characteristics, beliefs, medical condition, clinical history) that are relevant to considering how acceptable a placebogenic practice is. Does NOT include talk about how it is important to treat patients as individuals or to know one's patient very well (this is coded within placebogenic practice - therapeutic encounter considerations).	*The only observation I made just really when we started talking is that patients who may have that suspicious mind-set about drugs anyway tend to research side effect pretty effectively anyway. Again, if you know your patient you kind of reinforce the patient information leaflet about all the bad things that might happen.*<br><br>(GP Group 4)
Placebogenic practice – patient outcomes	The effects that participants think might flow from a particular placebogenic practice. These are effects on the patient as an individual (as opposed to their behaviour in	*F1: It's interesting because it could be that some people might think, oh you know, she cares about me and she wants to see me more, that's a positive thing,*

For peer review only

	relation to consulting and the doctor-patient encounter/relationship). Includes potential benefits and harms, of all types (e.g. includes psychological, physical, financial considerations). Also includes talk about the possible lack of effects of a placebogenic practice - i.e. when participants think that it would not work.	*F2: But it might also be a sign of added interest from –*<br><br>*F1: Yes, staying on top of it, you know, wanting to be on your shoulders, not fobbed off with a diary.*<br><br>(Patient Group 2)
Placebogenic practice – practitioner consequences	The effects that participants this might stem from a particular placebogenic practice. These are effects on the doctor as an individual and could include potential benefits and harms of all types.	[Quote from 'Monitoring' Scenario]<br>*I suppose I would use it for a few reasons as well. One would be both to better inform myself, because often patients come in with these vague symptoms and you're never quite sure what's actually happening, because they don't almost really know themselves. So actually getting them to sit down and actually write it out sometimes is very helpful, again, for them to establish, actually, this is the pattern of work. Maybe it isn't as bad as I thought it was because, of course, patients will often think the worst. So that can be really helpful. I think I probably also have used it as a procrastination technique every now and again, because I do think a medication will work but it just needs a bit more time.*<br><br>(GP Group 3)
Placebogenic practice – the practitioner considerations	Properties, characteristics, etc. of the practitioner that are relevant to considering the acceptability of a placebogenic practice. Includes discussion related to the practitioner's status, their qualifications and expertise, and their intentions	*Male 3: I'm not certain that last statement or sentence, 'if you tell him about it he'll be more likely to suffer from them' is true. I think there's a small cohort of patients where you tell them the side effects they will get it, and I could name a few patients where if I*

For peer review only - http://bmjopen.bmj.com/site/about/guidelines.xhtml

	guiding the placebogenic practice.	*say you're going to get cough with giving an ace inhibitor, they will cough. So those are the ones where arguably actually I would not tell them that you might get a cough about it. But I think on the whole patients –*<br><br>*Male 1: Would you be happy for one of your patients to come back to you nine months later and say, "I've had this cough for nine months," but they weren't aware it was side effect and they've had to live with that for nine months because you hadn't told them about it?*<br><br>*Male 3: I'd happily live with that, it's only a bit of a dry cough, it's not the end of the world, it's not going to kill –*<br><br>(GP Group 1)
Placebogenic practice – therapeutic encounter consequences	Talk about consequences of the placebogenic practice for the therapeutic encounter, the doctor-patient relationship, and future consultations. Includes talk about both positive and negative consequences.	*To me, it depends upon the frequency and the severity of the side effects. Because if they're rare and minor I would be completely comfortable with it, if they're serious or very frequent I'd be uncomfortable with it because you risk loss of trust, I think, from your patient if you don't tell them.*<br><br>(GP Group 1)
Placebogenic practice – therapeutic encounter considerations	Issues about the doctor-patient relationship in general and the consultation in particular that are deemed relevant to considering the acceptability of a placebogenic practice. Incudes discussion of the therapeutic encounter and its characteristics and how these might influence whether a particular practice is acceptable. Also includes	*I was going to say the same thing and it's the thing that is the doctor is the drug relationship, where you are using your ongoing built-up trust with the patient to have this effect, but if the effect doesn't actually happen or if the patient doesn't derive the benefit what then happens is you've lost some of that capital of the relationship. So*

For peer review only

	discussion of how a placebogenic practice itself represents a particular type of therapeutic encounter or promotes a particular style of consultation, relationship, etc.	*it is a judged thing in terms of how much you can use this on a day-to-day basis with individual patients. And it's a selective thing that you do use, well, you do use selectively. You use it when you need to, certainly not all the time.*<br><br>(GP Group 3)
Placebogenic practice acceptability depends on the belief system of the doctor and the patient	Participants speak of there needing to be agreement between the individual belief system of both the doctor and the patient for acceptability.	*This is a very different scenario to what we've had before where we've had someone who has effectively had no thoughts about something whatsoever and you can then say, 'This has really got every chance of working.' We are in a very different position. This is someone who has done their research; their belief system is such this isn't going to work. Well, you can't suddenly impose your belief system on their belief system, it doesn't work that way and if it did work that way actually, you know, the patient becomes very dependent and there's all sorts of stuff around that that you don't want. So, for me, this is about negotiating some form of change and when you go into the negotiating progress or process, you have to be aware that, actually, should I not succeed, I am going to have to go down one of the alternative avenues. I may not think they're as good but it's better that the patient takes a lesser treatment and takes it, rather than takes what is considered, in your opinion, to be the better treatment, with a sort of 'no-cebo' effect from the patient's perception; so, they're saying, 'Oh, I don't know if I like this, it's not going to work as well.' or they simply might take it*

For peer review only

		entirely against you and go and see a different doctor. So, I think it really is a very high risk strategy.<br><br>*(GP Group 2)*
Placebogenic practice is acceptable if it is not labelled as 'placebo'	Participants discuss that there is something about the label of placebo which governs acceptability.	*Well, I'm not particularly uncomfortable about that because, going back to the point about anti-depressants, I feel I am doing that every time I prescribe an anti-depressant for people with mild to moderate depression because I have a feeling that a lot of what gets better for the patient is either the rest of their psychological therapy, the time, the rearrangement of whatever social difficulties they happen to be in or whatever it is and probably a bit of placebo effect on the anti-depressant whereas an active – I don't think there's any active bit in the pill that's making a difference -*<br><br>(GP Group 2)
Placebogenic practice is not a placebo	Participants disagree that a particular scenario is an example of a placebo.	*[M1]: When you are talking about placebos, are we talking generally sugar pills, I mean that's what we tend to call them. I mean who's to say that an extra boost of sugar is not the kind of treatment that you want. And that it actually does make you better. It might trigger extra endorphins which might make you feel better.*<br><br>(Patient Group 4)
Placebogenic practice is undervalued in the art of medical practice	Discussion about the role of placebogenic practice as a skill in medical practice.	*Actually, I think we underutilise the placebo effect, because I think we could do more with it. So now I think it's a bit of a shame because, in fact, placebos do have some side-effects but, on the whole, less than many of the other tablets*

For peer review only

		we give people. … So I think there's a lost art, almost, that we're not utilising. The problem is it's being seen as deceptive to actually specifically give them a placebo that we don't believe has had any trial behind it to help in their instance. And I think that's where the problem comes about is it's actually our belief, whether it's true or not, or how it comes about for them. And so I suppose, for me, this is a grey area…So I suppose because medicine is so big now, we can't know everything about all the things that we prescribe. We are relying so heavily on other people's information…. So I would say there's a maybe there, and there's certainly times where I suppose I've prescribed antibiotics because the patient won't leave my consulting room so… but I'm sure it's not going to work. In fact, I'm sure I'm not treating a valid infection for them, but they've already spent 25 minutes arguing their case, so I'm going to give it to them. And it's a placebo, and I believe it's a placebo and they believe it's going to work which is the definition of a placebo, so yeah, I suppose, again, it's a vague, grey area. I'm not so hard and fast as everyone else.<br><br>*(GP Group 3)*
Placebogenic practice it is hard to decide	Participants cannot decide whether a placebogenic practice is acceptable or unacceptable. They can list advantages as well as disadvantages close together and find it difficult to come down on one side or the other.	*But I think you have got to be so careful who you target that at, so careful, because the breach of trust and that feeling of breach of trust, can have worse effects I think than the positive effect, if you see what I mean, so it is a balancing act.*

For peer review only

	There can be evidence of dilemmatic thinking and/or indecision.	(Patient group 1)

For peer review only

Reporting checklist for qualitative study.

Based on the SRQR guidelines.

Instructions to authors

Complete this checklist by entering the page numbers from your manuscript where readers will find each of the items listed below.

Your article may not currently address all the items on the checklist. Please modify your text to include the missing information. If you are certain that an item does not apply, please write "n/a" and provide a short explanation.

Upload your completed checklist as an extra file when you submit to a journal.

In your methods section, say that you used the SRQRreporting guidelines, and cite them as:

O'Brien BC, Harris IB, Beckman TJ, Reed DA, Cook DA. Standards for reporting qualitative research: a synthesis of recommendations. Acad Med. 2014;89(9):1245-1251.

		Reporting Item	Page Number
**Title**			
	#1	Concise description of the nature and topic of the study identifying the study as qualitative or indicating the approach (e.g. ethnography, grounded theory) or data collection methods (e.g. interview, focus group) is recommended	1

For peer review only

Abstract

	#2	Summary of the key elements of the study using the abstract format of the intended publication; typically includes background, purpose, methods, results and conclusions	2

Introduction

Problem formulation	#3	Description and signifcance of the problem / phenomenon studied: review of relevant theory and empirical work; problem statement	3
Purpose or research question	#4	Purpose of the study and specific objectives or questions	3

Methods

Qualitative approach and research paradigm	#5	Qualitative approach (e.g. ethnography, grounded theory, case study, phenomenolgy, narrative research) and guiding theory if appropriate; identifying the research paradigm (e.g. postpositivist, constructivist / interpretivist) is also recommended; rationale. The rationale should briefly discuss the justification for choosing that theory, approach, method or technique rather than other options available; the assumptions and limitations implicit in those choices and how those choices influence study conclusions and transferability.	4-6

For peer review only

		As appropriate the rationale for several items might be discussed together.	
Researcher characteristics and reflexivity	#6	Researchers' characteristics that may influence the research, including personal attributes, qualifications / experience, relationship with participants, assumptions and / or presuppositions; potential or actual interaction between researchers' characteristics and the research questions, approach, methods, results and / or transferability	3
Context	#7	Setting / site and salient contextual factors; rationale	4
Sampling strategy	#8	How and why research participants, documents, or events were selected; criteria for deciding when no further sampling was necessary (e.g. sampling saturation); rationale	4
Ethical issues pertaining to human subjects	#9	Documentation of approval by an appropriate ethics review board and participant consent, or explanation for lack thereof; other confidentiality and data security issues	3-4
Data collection methods	#10	Types of data collected; details of data collection procedures including (as appropriate) start and stop dates of data collection and analysis, iterative process, triangulation of sources / methods, and modification of procedures in response to evolving study findings; rationale	4-6

For peer review only

Data collection instruments and technologies	#11	Description of instruments (e.g. interview guides, questionnaires) and devices (e.g. audio recorders) used for data collection; if / how the instruments(s) changed over the course of the study	4-6
Units of study	#12	Number and relevant characteristics of participants, documents, or events included in the study; level of participation (could be reported in results)	6
Data processing	#13	Methods for processing data prior to and during analysis, including transcription, data entry, data management and security, verification of data integrity, data coding, and anonymisation / deidentification of excerpts	6
Data analysis	#14	Process by which inferences, themes, etc. were identified and developed, including the researchers involved in data analysis; usually references a specific paradigm or approach; rationale	6
Techniques to enhance trustworthiness	#15	Techniques to enhance trustworthiness and credibility of data analysis (e.g. member checking, audit trail, triangulation); rationale	6
**Results/findings**			
Syntheses and interpretation	#16	Main findings (e.g. interpretations, inferences, and themes); might include development of a theory or model, or integration with prior research or theory	7

For peer review only

Links to empirical data	#17	Evidence (e.g. quotes, field notes, text excerpts, photographs) to substantiate analytic findings	7
**Discussion**			
Intergration with prior work, implications, transferability and contribution(s) to the field	#18	Short summary of main findings; explanation of how findings and conclusions connect to, support, elaborate on, or challenge conclusions of earlier scholarship; discussion of scope of application / generalizability; identification of unique contributions(s) to scholarship in a discipline or field	11-12
Limitations	#19	Trustworthiness and limitations of findings	12
**Other**			
Conflicts of interest	#20	Potential sources of influence of perceived influence on study conduct and conclusions; how these were managed	1
Funding	#21	Sources of funding and other support; role of funders in data collection, interpretation and reporting	1

The SRQR checklist is distributed with permission of Wolters Kluwer © 2014 by the Association of American Medical Colleges. This checklist was completed on 04. June 2019 using https://www.goodreports.org/, a tool made by the EQUATOR Network in collaboration with Penelope.ai

For peer review only
